# Prognostic model of kidney renal clear cell carcinoma using aging-related long noncoding RNA signatures identifies THBS1-IT1 as a potential prognostic biomarker for multiple cancers

**DOI:** 10.18632/aging.204949

**Published:** 2023-09-13

**Authors:** Yi-Fan Tang, Yu-Zhi Wang, Gui-Biao Wen, Jian-Jun Jiang

**Affiliations:** 1 Department of Nephrology, Shenzhen Traditional Chinese Medicine Hospital, The Fourth Clinical Medical College of Guangzhou University of Chinese Medicine, Shenzhen, China; 2 Department of Urology, Shenzhen Traditional Chinese Medicine Hospital, The Fourth Clinical Medical College of Guangzhou University of Chinese Medicine, Shenzhen, China

**Keywords:** aging, pan-cancer, biomarker, machine learning, prognosis

## Abstract

Aging is responsible for the main intrinsic triggers of cancers; however, the studies of aging risk factors in cancer animal models and cancer patients are rare and insufficient to be represented in cancer clinical trials. For a better understanding of the complex regulatory networks of aging and cancers, 8 candidate aging related long noncoding RNAs (CarLncs) identified from the healthy aging models, centenarians and their offsprings, were selected and their association with kidney renal clear cell carcinoma (KIRC) was explored by series of cutting edge analyses such as support vector machine (SVM) and random forest (RF) algorithms. Using data downloaded from TCGA and GTEx databases, a regulatory network of CarLncs-miRNA-mRNA was constructed and five genes within the network were screened out as aging related feature genes for developing KIRC prognostic models. After a strict filtering pipeline for modeling, a formula using the transcript per million (TPM) values of feature genes “LncAging_score = 0.008* MMP11 + 0.066* THBS1-IT1 + (-0.014)* DYNLL2 + (-0.030)* RMND5A+ 0.008* PEG10” was developed. ROC analysis and nomogram suggest our model achieves a great performance in KIRC prognosis. Among the 8 CarLncs, we found that *THBS1-IT1* was significantly dysregulated in 12 cancer types. A comprehensive pan-cancer analysis demonstrated that *THBS1-IT1* is a potential prognostic biomarker in not only KIRC but also multiple cancers, such as LUSC, BLCA, GBM, LGG, MESO, PAAD, STAD and THCA, it was correlated with tumor microenvironment (TME) and tumor immune cell infiltration (TICI) and its high expression was related with poor survival.

## INTRODUCTION

Aging is an unavoidable age-associated physiological decline caused by a progressive dysregulation of certain cellular and organismal processes [1]. Cancer is the leading cause of death worldwide and was regarded as an aging-related disease as most individuals are diagnosed after they reach fifty [2]. Mortality statistics also reflected the age dependency of cancers. According to the data from Global Burden of Disease Study (GBD 2019), elders ≥70 years old had the largest burden of cancer mortality [3]. Moreover, the underlying biological process of cancers hallmarks are overlapped with aging such as corrupted proteostasis, genomic instability, telomere attrition, aggravated inflammation and heightened cellular senescence [2]. Noteworthy, renal cell carcinoma (RCC) has a high occurrence in aged 65 years or older and an average first diagnosed age at 64, making it one of cancer types strongly correlated with aging [4]. As such aging and cancer are considered as two tightly interconnected biological phenomena; however, the studies of aging risk factors in cancer animal models and cancer patients are rare and insufficient to be represented in cancer clinical trials.

Long noncoding RNA (lncRNA) represents a large group of noncoding RNA that is generally classified as transcripts ≥ 200bp without coding potential [5, 6]. With the significantly important and indispensable roles in various biological processes and dynamic expression patterns in tissues and cells, lncRNAs have been implicated promising diagnostic and prognostic biomarkers in human diseases especially in cancers [7]. In our previous study of deciphering lncRNA features in 171 individuals from centenarian (CEN) families, 8 candidate aging related lncRNAs (CarLncs) were identified including *THBS1-IT1*, *THBS1-AS1*, *DCHS1-AS1*, *LINC01871*, *LEF1-AS1-201*, *WDR11-AS1-201* and *GRAPLDR.* Comparing with normal elders, these 8 CarLncs displayed consistent and significant dysregulation in the healthy aging models – CENs and their offsprings (8). *In vitro* functional assays validated that these lncRNAs especially *THBS1-IT1* and *THBS1-AS1* are involved in cellular senescence process and may act as anti-cellular aging protective elements in the healthy aging of CENs [8]. *THBS1-AS1* was positively related with cardiac fibrosis and regulates the expression of Tgfbr1 through sponging with mir-221/222 [9]. To gain a better knowledge of potential function of aging related factors in cancer ontogeny and cancer treatment, we analyzed the expression signatures of 8 CarLncs in kidney renal clear cell carcinoma (KIRC) as KIRC is a histopathological subtype of RCC that represents 80% to 90% of all RCC tumors. Analyses such as co-efficient correlation analysis, Wilcoxon test, random forest (RF) algorithms and support vector machine (SVM) were used for screening and validating the prognostic risk-related factors. Five genes were finally selected and employed for KIRC prognosis, among which, *THBS1-IT1* was further evaluated for a pan-cancer analysis as it exhibited significant dysregulation in 12 cancers. Our results demonstrated that *THBS1-IT1* possesses potential capacity for the prognosis of multiple cancers. Taken together, this study provided new insights into the complicated and underlying associations between aging and cancers.

## MATERIALS AND METHODS

### Data availability

The KIRC cohort data which contain 541 tumor samples and 72 para-cancer samples were downloaded from TCGA data portal (https://portal.gdc.cancer.gov). Gene expression profiles, survival values, clinicopathological information and somatic mutation data of 33 tumor types, as well as the gene expression levels of 31 tissues in GTEx database, were obtained from UCSC Xena browser (https://xena.ucsc.edu).

### Identification of AR-subgroup using the expression profiles of CarLncs

The Wilcoxon test was used to determine the variation of 8 CarLncs’ expression among 541 KIRC patients. Using their expression profiles, we employed consensus clustering to define distinct aging-related subgroups (AR-subgroup) by k-means algorithms in R platform [10]. The quantity and consistency of clusters were evaluated by “ConsensusClusterPlus” R package with default parameters [11]. To ensure the stability of the categories, 50 iterations and 80% resampling rate Pearson correlation analysis was performed.

### Characterization of TME in two AR-subgroups

Principal component analysis (PCA) analysis was performed to demonstrate the internal distribution of each AR-subgroup. To investigate the differences of biological function of 8 CarLncs, gene set variation analysis (GSVA) was conducted using the KEGG gene set (c2.cp.kegg.v7.4) [12]. The infiltrating fractions of immune cells were identified via single-sample gene set enrichment analysis (ssGSEA) algorithm with default parameters [10].

### Correlation between AR-subgroups and clinical characteristics

To determine the clinical significance of AR-subgroups, we investigated the association among molecular patterns, clinical features, and survival outcomes. Age, gender, grade, stage, T-stage, M-stage, and N-stage were included as the clinical variables.

### Comparisons of OS between AR-subgroups in KIRC

Kaplan–Meier (KM) analysis was used to evaluate the difference of OS between two AR-subgroups via “survival” and “survminer” R packages.

### Construction of the CarLnc-miRNA-mRNA network

The CarLnc-miRNA-mRNA network was conducted following an optimized method described in previous studies [13, 14]. Firstly, differentially expressed mRNAs (DE-mRNA) and miRNAs (DE-miRNA) between AR-subgroups were identified by “limma” R package with parameters of |log_2_(fold change) | > 0 and p value < 0.05. MiRNA targets that potentially bound to CarLncs (miRNA-Tgt) were predicted by lncBase Predicted v.2 database [15]. Subsequently, three databases include miRDB [16], miRTarBase [17] and TargetScan [18] were used to predict mRNA targets of miRNA-Tgt (mRNA-Tgt). Finally, only the overlapped miRNA/mRNA between miRNAs-Tgt and DE-miRNAs/mRNAs-Tgt and DE-mRNAs were retained and their interplays with CarLncs were visualized using Cytoscape 3.9.0 software [19].

### Functional enrichment analysis

Gene Ontology (GO) [20] and Kyoto Encyclopedia of Genes and Genomes (KEGG) [21] analyses were conducted by “clusterProfiler” R package [22].

### Screening feature genes of AR-subgroups

Feature genes are genes that can be used to distinguish the two AR-subgroups. The molecules within CarLnc-miRNA-mRNA network were used to determine the feature genes by random forest (RF) algorithm and support vector machine - recursive feature elimination (SVM-RFE). At the first step, “e1071” [23] and “caret” [24] R packages were utilized for screening the optimal gene sets as the signature of AR-Subgroups using SVM-RFE method [25] as SVM-RFE is an iterative backward elimination procedure for feature selection [24]. Secondly, “randomForest” R package was used to construct the optimal random forest classification model (ntree=500), and then to evaluate the correlation weight of genes within the network to characterize the effect of the gene on aging-related classification (indicator “MeanDecreaseGini”) [26]. Finally, the intersected genes between the optimal gene set screened by the SVM-RFE algorithm and the top-ranked genes with “MeanDecreaseGini” ≥2.0 were regarded as aging-related feature genes in KIRC patients.

### Developing the KIRC prognostic model using aging-related feature genes

We removed samples without clinical survival data and clinicopathological information, a total of 514 samples were retained. They were randomly divided into training (n=345) and validation (n=169) groups. Multivariate stepwise COX regression analysis (multi-Cox) was performed on the aging-related feature genes of KIRC patients in the training dataset to construct the aging-related prognostic model which was visualized by forestplot. Model with the smallest Akaike information criterion (AIC) value and the highest C-index was selected as the final prognostic model, CarLncs-based aging score (LncAging_score) was developed to represent the model and it was calculated by the following algorithm:


$$\text{LncAging\_score}=\sum {\text{coef}}_{i}\times X_{i}$$

where *X_i_* is the expression of the variable in the aging-related model and coef*_i_* is the regression coefficient of the variable.

We used the median value of LncAging_score of training group as a threshold, and separately divided the training, the validation and the entire samples into high (≥ median LncAging_score) and low (< median LncAging_score) groups. Survival curves of OS and progression-free interval (PFI), ROC curves, and risk plots were visualized to evaluate the predictive validity of the CarLncs-based aging prognostic model by examining the differences between high and low LncAging_score groups.

### Establishment of a predictive nomogram

The “rms” R package [27] was used to depict a nomogram to provide clinical predictions for KIRC patients using their LncAging_scores and clinicopathological characteristics such as age, gender, stage, 1-, 3-, and 5-year OS. Next, we performed calibration curve, decision curve analysis (DCA) and concordance index (C-index) to verify the clinical reliability of the established nomogram. Finally, we performed Kaplan–Meier (KM) analysis and ROC curve analysis on the risk score of the nomogram (Nomo-risk) to evaluate the accuracy of prognostic prediction.

### Pan-cancer analysis of the association among *THBS1-IT1*, prognosis and clinical phenotype

Survival and clinical phenotype data were downloaded from TCGA. Four indicators include disease-specific survival (DSS), overall survival (OS), progression-free interval (PFI), and disease-free interval (DFI) were selected to explore the correlation between *THBS1-IT1* expression and the prognostic status of patients. The effects of *THBS1-IT1* expression on survival was evaluated by KM analysis and log-rank test using R packages “survival” and “survminer” as well as COX analysis by “survival” and “forestplot” methods [28].

Two clinical phenotypes consisting of tumor stage and age were selected and their relationships with *THBS1-IT1* expression were explored. Patients were divided into two groups based on their ages, with 65 years old as the threshold. The correlation analyses were conducted using “limma” and “ggpubr” R-packages [29].

### Correlation analysis of *THBS1-IT1* with tumor mutation burden and tumor microsatellite instability in pan-cancer

Tumor Mutation Burden (TMB) scores were calculated following a published method which utilized Perl scripts for correction via dividing by the total length of exons [30]. Tumor Microsatellite Instability (MSI) scores in all samples were calculated using the somatic mutation data downloaded from TCGA (https://tcga.xenahubs.net). The correlations between *THBS1-IT1* expression and TMB/MSI scores were analyzed by Spearman’s rank correlation coefficient.

### Evaluation of the association between *THBS1-IT1* and immunity in pan-cancer

The degree of infiltration of stromal or immune cells into tumors was assessed by estimating the immune scores and stromal scores using “estimate” and “limma” R packages as previously described [31]. The associations between *THBS1-IT1* expression and the two scores were calculated by Spearman’s correlation co-efficient analysis [32]. Additionally, TIMER (Tumor Immune Estimation Resource) database [33] was utilized to analyze the infiltration of immune cells including Dendritic cells, B cells, Neutrophils, Macrophages, CD4+ T cells and CD8+ T cells in tumor tissues.

### Statistical analysis

α = 0.05 was taken as the significance standard. Comparisons between groups were performed by Wilcoxon rank-sum test, independent samples T-test, chi-square test, Fisher’s exact test, etc. The correlation coefficient was analyzed by Spearman’s or Pearson’s correlation co-efficient analysis. All the R packages used in this study were operated by R (version 4.2.1).

### Data availability statement

All the raw data in this study are available from the corresponding author upon reasonable request.

## RESULTS

### Classification of aging related subgroups (AR-subgroups) in KIRC using CarLncs

**Figure 1:**
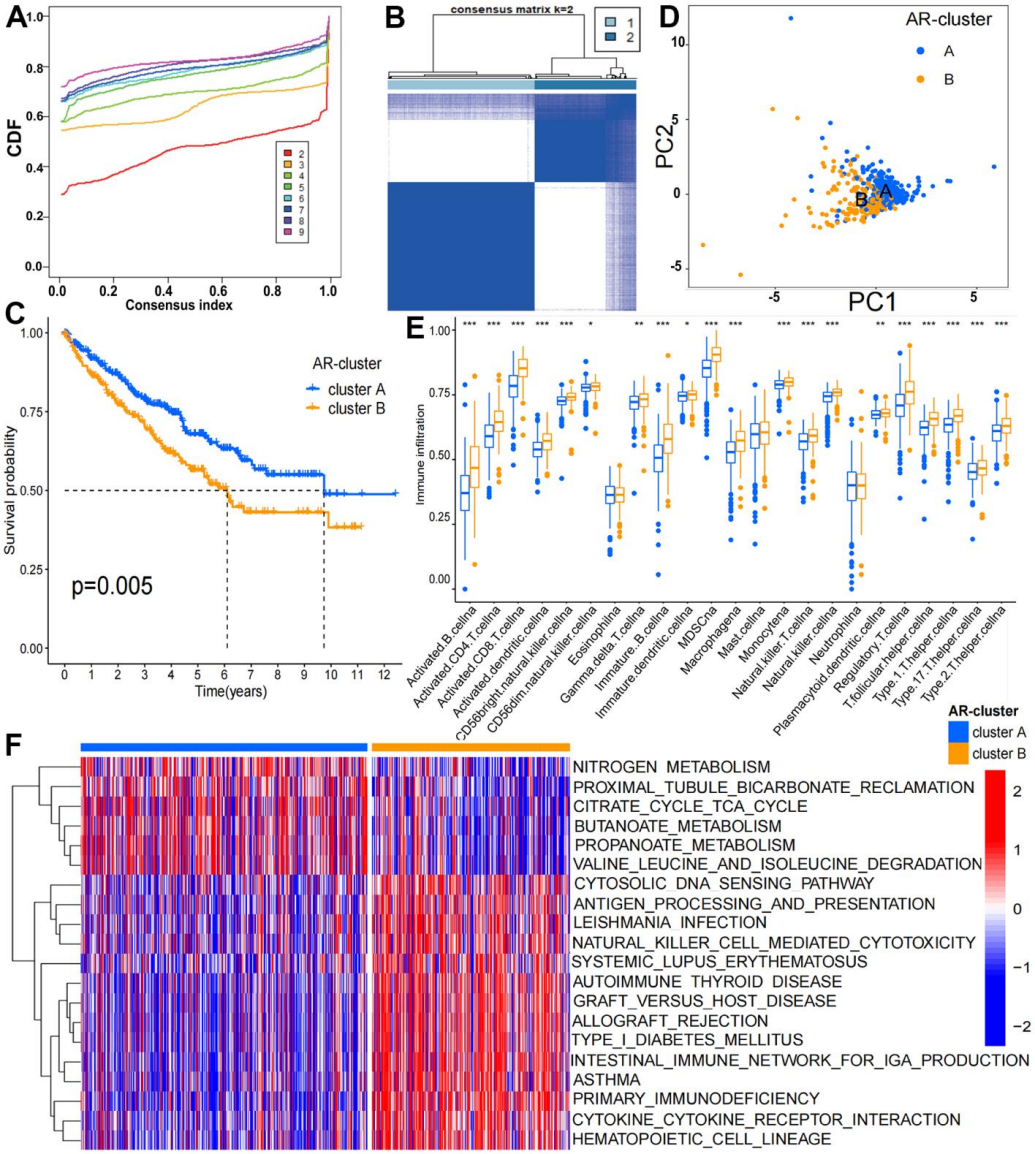
**Clinicopathological and biological features of the two ARL subgroups divided by consistent clustering.** (**A**) Consensus matrix CDFs from 2 to 9; (**B**) Two distinct clusters (k = 2) and their correlation area were identified by consensus matrix heatmap; (**C**) OS between cluster A and B is significantly different by KM analysis; (**D**) PCA analysis showed the transcriptome of the two subgroups is apparently different; (**E**) The abundance of 19 infiltrating immune cells is significantly different in two KIRC subgroups; (**F**) Biological pathways of the two distinct subgroups by GSVA analysis (p < 0.05 *; p < 0.01 **; p < 0.001 ***).

The 8 CarLncs identified by our previous study were used as signatures genes to investigate their potential roles in KIRC (5). To gain the potential expression patterns and roles of 8 CarLncs in fields other than aging and cancer, we added the expression profiles of 8 CarLncs through the comprehensive human lncRNA database – LncExpDB (https://ngdc.cncb.ac.cn/lncexpdb/). Probably due to the genome annotation files used by LncExpDB database (Hg38) was different from our version (Hg19), only 5 lncRNAs were detected. It showed that the 5 CarLncs (*THBS1-IT1*, *DCHS1-IT1*, *LINC01871*, *LEF1-AS1-201* and *WDR11-DT-201*) exhibit dynamic expression from early organogenesis to adulthood and display extinct tissue specificity and circadian rhythmicity (Supplementary Figure 1). For a better understanding of the aging related expression patterns in KIRC, a total of 514 tumor patients with both expression profiles and survival/clinicopathological data were enrolled to reveal the relationship between aging and tumorigenesis (Supplementary Table 1). The flowchart of this study was shown in Supplementary Figure 2. Firstly, we compared the expression levels of 8 CarLncs between tumor and adjacent tissues and found that *THBS1-IT1*, *LINC01871*, *LEF1-AS1-201*, *CCL3-AS1-202*, *GRAPLDR* and *WDR11-DT-201* were significantly differentially expressed (Supplementary Figure 3, P<0.05), suggesting these 6 lncRNAs are related with both aging and KIRC. Using the expression profiles of the 6 dysregulated CarLncs (DS-CarLncs) as the candidate signatures, patients (n=514) were then subjected for classification using consensus clustering analysis. K-means clustering showed that the optimal clustering variable was 2 (Figure 1A) and patients were classified into two clusters (Figure 1B). A significant difference (P<0.05) of overall survival (OS) was observed between the two subgroups by KM analysis (Figure 1C). PCA analysis was used to display the intergroup distribution, which further confirmed that two clusters (cluster A and B) were well generated by CarLncs expression data (Figure 1D). As shown in Supplementary Figure 4, the comparisons of the expression level of 8 CarLncs and clinicopathological variables also suggested a substantial difference between these two groups. These results suggested the enrolled KIRC patients may possibly possess two different models of aging.

We noticed that the abundance of 24 immune cells between two clusters was substantially different (Figure 1E). The enrichment levels of 19 immune cell types were remarkably higher in cluster B (n = 211) than cluster A (n= 303), which include activated B cell, macrophage, activated CD4 T cell, MDSC, activated CD8 T cell, NK T cell, activated dendritic cell, NK cell, CD56 bright natural killer cell, type 17 T helper cell, CD56 dim natural killer cell, gamma delta T cell, type 1 T helper cell, immature B cell, immature dendritic cell, monocyte, regulatory T cell, T follicular helper cell, plasmacytoid dendritic cell and type 2 T helper cell. GSVA analysis suggested that cluster B was enriched in immune-associated pathways including intestinal immune network for IgA production (hsa04672), primary immunodeficiency (hsa05340) and autoimmune thyroid disease (hsa05320) while cluster A (n= 303) was enriched in metastasis-associated pathways consisting pyruvate metabolism (hsa00620), fatty acid metabolism and histidine metabolism (hsa01212) (Figure 1F and Supplementary Table 2). The results indicated the tumor immune environment of cluster A and B may be different.

### Construction the CarLnc-miRNA-mRNA network

**Figure 2:**
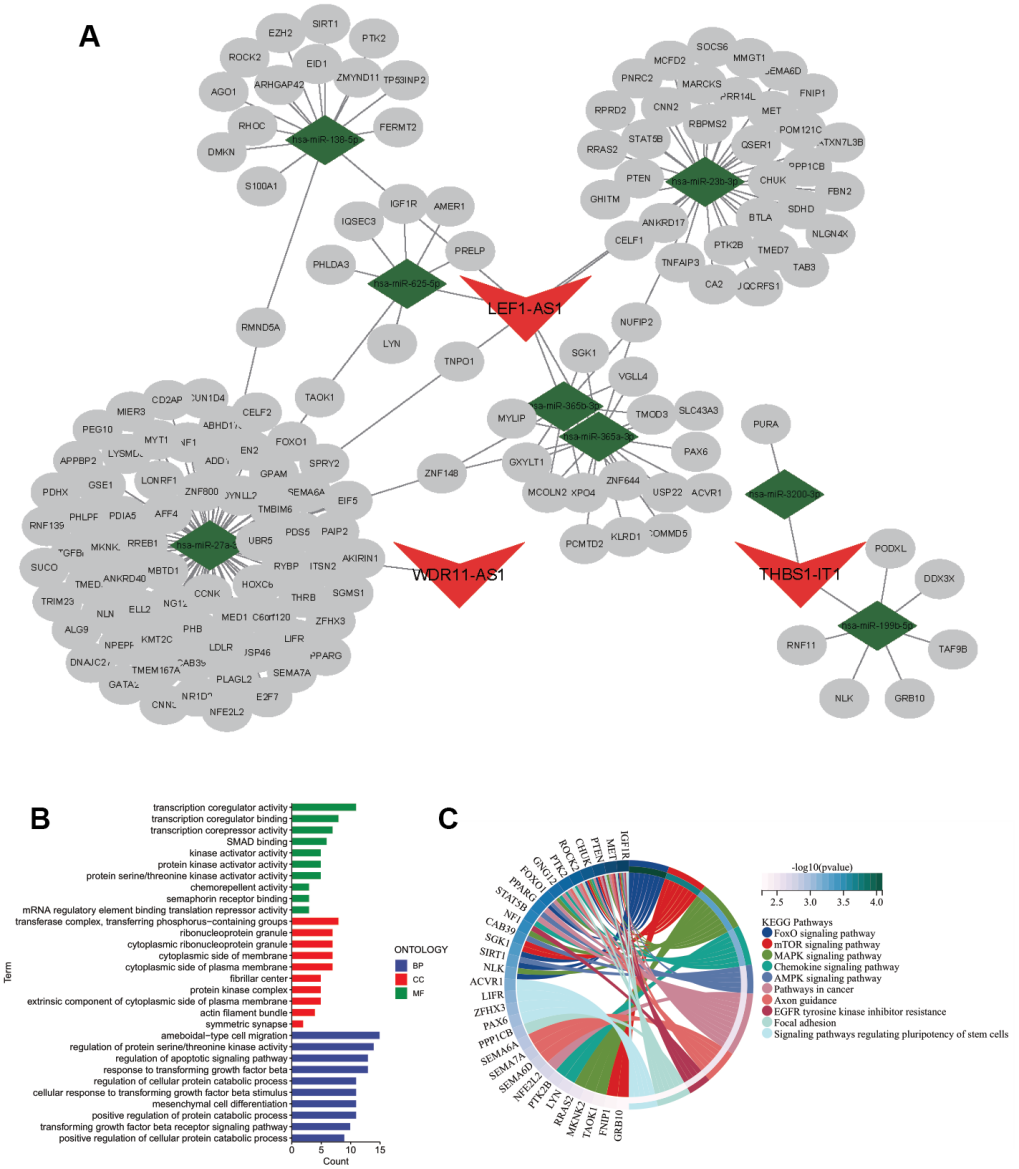
**Regulatory network of CarLnc-miRNA-mRNA.** (**A**) Visualization of CarLnc-miRNA-mRNA network by Cytoscape; (**B**) The top GO terms of the 148 mRNAs within the network by gene enrichment analysis; (**C**) The top enriched pathways of 148 mRNAs by KEGG enrichment analysis.

To elucidate the potential regulatory function of CarLncs in aging and KIRC tumorigenesis, a CarLnc-miRNA-mRNA network (Figure 2A) was constructed. Firstly, the potential miRNA targets of the 6 DS-CarLncs (miRNA-Tgt) and the candidate mRNA targets of miRNA-Tgt (mRNA-Tgt) were predicted using online web tools. Considering the AR-subgroups of KIRC were generated based on the expression patterns of 6 DS-CarLncs, differentially expressed mRNA (DE-mRNA) and miRNA (DE-miRNA) between two subgroups were identified as these mRNAs/miRNAs may also associated with these two aging models. Only 8 miRNAs are overlapped between DE-miRNA and miRNA-Tgt which include *miR-199b-5p*, *miR-3200-3p*, *miR-138-5p*, *miR-23b-3p*, *miR-365a-3p*, *miR-365b-3p*, *miR-625-5p* and *miR-27a-3p*. These 8 overlapped miRNAs are potential targets of 3 DS-CarLncs, which is *THBS1-AS1*, *LEF1-AS1-201* and *WDR11-DT-201*. Comparing DE-mRNA and mRNA-Tgt,148 mRNAs were intersected and were retained for further analysis. Finally, a lncRNA-miRNA-mRNA regulatory network was constructed using the 148 mRNAs, the 8 miRNA and the 3 CarLnc (Figure 2A and Supplementary Table 3).

As the 148 mRNAs were differentially expressed between 2 AR-subgroups of KIRC, they were also predicted as targets of CarLncs related miRNAs; therefore, we hypothesized that they may be involved in both aging process and KIRC. To explore their potential function and association with aging/KIRC, functional enrichment analysis was employed. GO analysis showed that they were primarily associated with protein serine/threonine kinase activator activity (GO:0043539), serine/threonine protein kinase complex (GO:1902554) and positive regulation of protein catabolic process (GO:0045732) (q<0.05, Figure 2B). The pathway enrichment analysis revealed that they were associated with FoxO signaling pathway (hsa04068), MAPK signaling pathway (hsa04010) and mTOR signaling pathway (hsa04150) (q<0.05, Figure 2C).

### Development of KIRC prognostic model using aging-related feature genes

**Figure 3:**
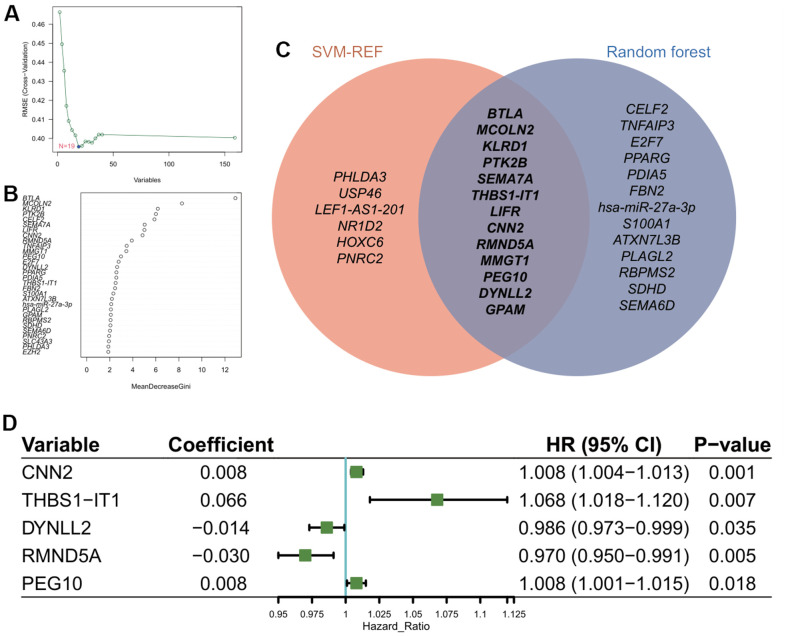
**Construction of prognostic model using feature genes.** (**A**) Identification of optimal gene set for the signature of CarLncs subgroups based on the SVM-RFE algorithm. (**B**) Top thirty genes with the highest “MeanDecreaseGini” for the optimal RF model. (**C**) Venn diagram of the overlapped genes of the two algorithms; (**D**) Independent prognostic factors for developing the aging related prognostic model were identified by Cox HR model.

SVM-RFE and RF algorithms were utilized to reduce the dimensionality of 159 genes within the network for feature genes identification. We obtained 19 genes with an optimal RMSE (Figure 3A) by SVM-RFE and 26 genes with “MeanDecreaseGini” index >2.0 by RF (Figure 3B). Among the two gene sets, 13 are overlapped in which *THBS1-IT1* was included (Figure 3C). KIRC patients (n=514) were randomly assigned into the training cohort (n=345) and the validation cohort (n=169). Four selected clinical terms (age, gender, grade and stage) of the training and validation groups were subjected to Chi-square tests to evaluate the randomness between two AR-subgroups and non-significant difference was observed (Supplementary Table 4). After steps of filtering by multi-Cox analysis, five genes were selected as the variables for model construction. We developed CarLncs-based aging score (LncAging_score) prognostic model to predict prognosis of KIRC patients. By multivariate stepwise regression (Figure 3D), an optimal prognostic model using the TPM values (transcript per million) of 5 selected feature genes was constructed using the training dataset (Figure 3D), the formula was described as follows:

LncAging_score = 0.008* MMP11 + 0.066* THBS1-IT1 + (-0.014)* DYNLL2 + (-0.030)* RMND5A+ 0.008* PEG10.

**Figure 4:**
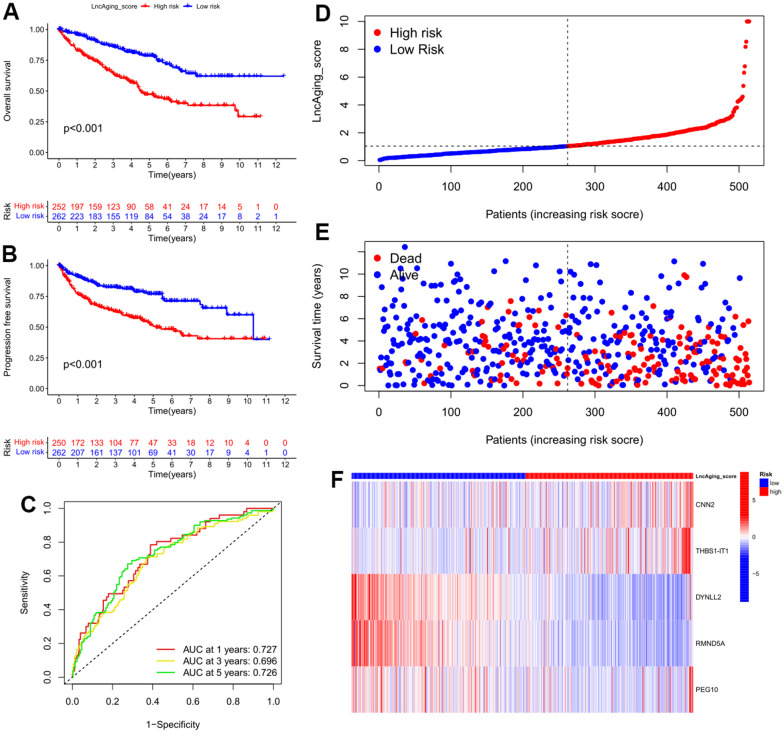
**Features of LncAging_score as the prognostic model in the training cohort.** (**A**) The sensitivity and specificity of 1-, 3-, and 5-year overall survival in two groups were predicted by ROC curves according to the LncAging_score; OS (**B**) and PFI (**C**) were compared between two groups by KM analysis; The distribution of LncAging_score (**D**) and survival status (**E**) in low- and high-risk patients was displayed by ranked dots and scatter plots; (**F**) The expression levels of 5 selected prognostic genes in two groups.

Based on the aging-related prognostic model, *CNN2*, *THBS1-IT1*, *DYNLL2*, *RMND5A* and *PEG10* were predicted as independent prognostic factors (Figure 3D, P<0.05), among which *CNN2*, *THBS1-IT1* and *PEG10* are risk factors (Figure 3D, hazard ratio (HR) >1). In the training cohort, Kaplan-Meier analysis indicated that low-risk patients had a better OS or PFI than high-risk patients (Figure 4A, 4B), and the AUCs of 1-, 3-, and 5-years OS were 0.727, 0.696, and 0.726, respectively (Figure 4C). The risk plot showed that OS was negatively while mortality was positively related with LncAging_score (Figure 4D, 4E). The correlation between risk power and gene expression level of 5 selected genes was shown in Figure 4F.

To assess the predictive robustness of the model, the LncAging_scores of validation group individuals were calculated. Using the median value LncAging_score of training cohort as the threshold, patients of validation group, training group as well as the entire cohort were separately assigned into 2 subgroups, which are low-risk (LncAging_score < median value) and high-risk (LncAging_score ≥ median value). Meanwhile, a superior OS was found in low-risk than high-risk patients by survival analysis and the 1-, 3-, and 5-year survival probability predicted by AUC value implied that LncAging_scores had a great performance in assessing the prognosis of KIRC patients (Supplementary Figure 5). The prognostic independence of LncAging_score and four clinical factors including age, disease grading, stage in the entire cohort demonstrated that LncAging_score, age, grade and stage are independent prognostic factors of KIRC (P<0.05, HR>1) (Supplementary Figure 6).

### Nomogram analysis suggests LncAging_score is a good model for KIRC prognostic

**Figure 5:**
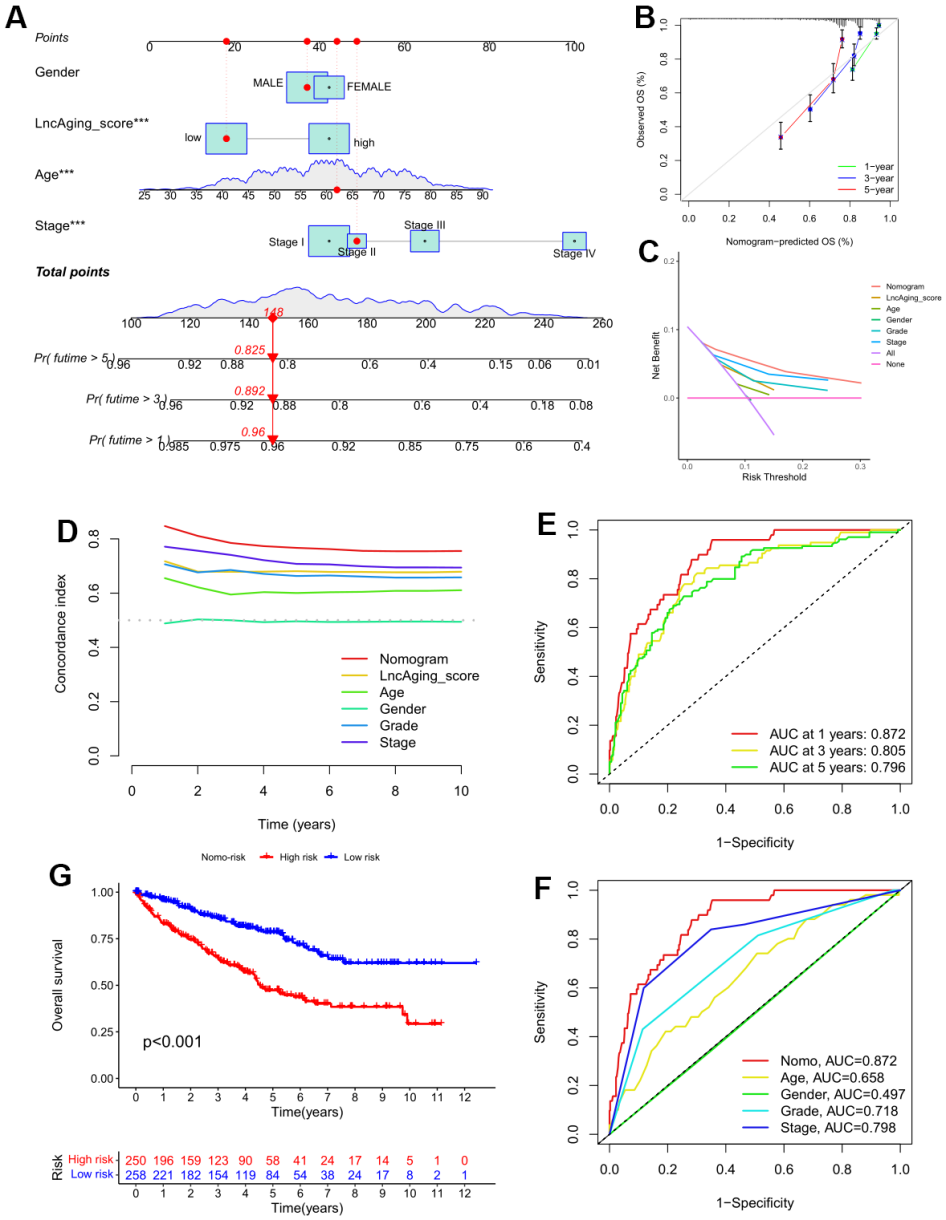
**A nomogram was constructed for validating the prognostic model in the entire KIRC cohort.** (**A**) The 1-, 3-, and 5-year OS in KIRC patients of the entire cohort was predicted by Nomogram; (**B**) Calibration curve of the nomogram and actual OS; Comparisons of DCA curve (**C**) and C-index (**D**) among the nomogram, LncAging_score model and 5 clinical variables for 1−year OS prediction; (**E**) ROC curves of the Nomo-risk at 1-, 3-, and 5-year; (**F**) ROC curves of Nomogram and 4 clinical variables for 1-year risk prediction; (**G**) KM survival analysis for KIRC patients with high- or low-Nomo-risk.

To further assess the significance of LncAging_score in KIRC prognosis, LncAging_score together with three clinical parameters (age, sex and stage information) were incorporated as variables to predict 1-, 3-, and 5-year OS for the entire KIRC cohort by nomogram (Figure 5A). Calibration curves of the established nomogram presented good consistency between actual observations and predicted values (Figure 5B). LncAging_score combined with the three clinical factors presented the best net benefits than single variable for predicting prognosis (Figure 5C, 5D). The AUC values of Nomo-risk at 1-, 3-, and 5-year risks were presented in Figure 5E. ROC curves of the nomogram as well as four clinical factors (age, gender, grade and stage) for 1-year risk were shown in Figure 5F. The range of AUC values of Nomo-risk was 0.796-0.872, which suggested that our nomogram achieves a good degree of accuracy for KIRC prognosis. Additionally, survival analysis based on the risk score of the nomogram showed that high Nomo-risk is related with poor prognosis (Figure 5G, P < 0.001).

### *THBS1-IT1* displayed abnormal expression in multiple cancer types

**Figure 6:**
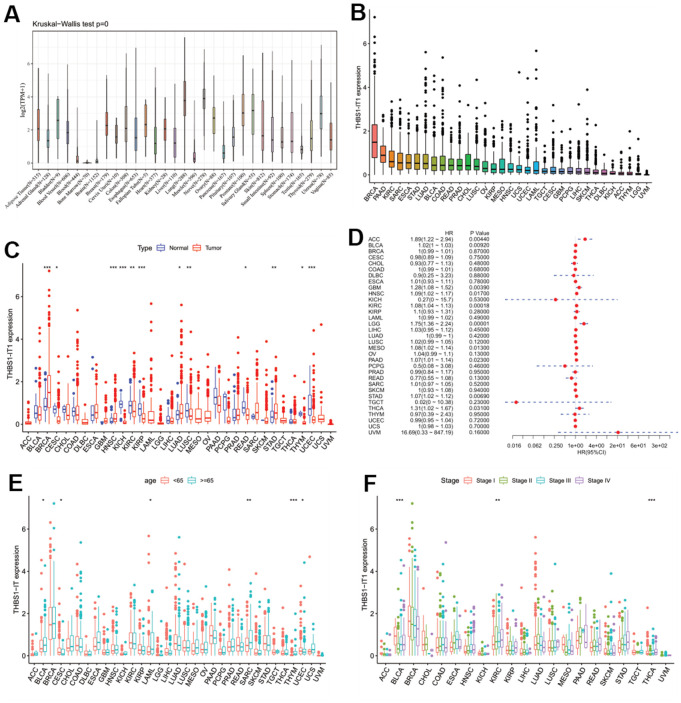
**Pan-cancer analysis of *THBS1-IT1*.** (**A**) Distribution of *THBS1-IT1* expression in normal tissues; (**B**) *THBS1-IT1* expression in 33 cancers; (**C**) Comparisons of *THBS1-IT1* expression between tumor and normal samples suggested it was dysregulated in 12 cancer types; (**D**) The association between *THBS1-IT1* expression and OS in 33 types of tumors by forest plot; (**E**) Correlation between *THBS1-IT1* gene expression and age; (**F**) The relevance of tumor stage and *THBS1-IT1* expression. *P < 0.05, **P < 0.01, ***P < 0.001.

Our previous study has implicated that overexpression of *THBS1-IT1* affects endoplasmic reticulum (ER) stress signaling and decreases p53 express [8]. Considering ER stress and p53 are broadly involved in the ontogeny and progression of pan-cancer, we hypothesized that *THBS1-IT1* may associate with the tumorigenesis of multiple cancers other than KIRC. Gene expression data of *THBS1-IT1* in 31 normal tissues and 33 types of tumor tissues were separately acquired from GTEx and TCGA databases, it showed *THBS1-IT1* is a widely expressed lncRNA gene in the majority of normal tissues (n=31) and all the tumor types (Figure 6A). Breast invasive carcinoma (BRCA), pancreatic adenocarcinoma (PAAD) and KIRC ranked the top three tumor types that expressed highest *THBS1-IT1* (Figure 6B)*.* Comparing with normal controls, *THBS1-IT1* showed significant different expression in BRCA, cervical squamous cell carcinoma and endocervical adenocarcinoma (CESC), head and neck squamous cell carcinoma (HNSC), KIRC, kidney chromophobe (KICH), kidney renal papillary cell carcinoma (KIRP), lung squamous cell carcinoma (LUSC), lung adenocarcinoma (LUAD), stomach adenocarcinoma (STAD), rectum adenocarcinoma (READ), thymoma (THYM) and uterine corpus endometrial carcinoma (UCEC) (Figure 6C, P<0.05).

### Evaluation of the predictive potential of *THBS1-IT1* in pan-cancer prognosis

To evaluate the effects of *THBS1-IT1* expression level on tumor prognosis, a series of survival association analyses using four indicators including overall survival (OS), disease-specific survival (DSS), disease-free interval (DFI) and progression-free interval (PFI) were performed in each cancer type by Cox proportional hazards model (Cox) and KM survival analysis. Cox-OS analysis showed that *THBS1-IT1* expression levels were associated with OS in adrenocortical carcinoma (ACC), bladder urothelial carcinoma (BLCA), glioblastoma multiforme (GBM), KIRC, brain lower grade glioma (LGG), mesothelioma (MESO), PAAD, STAD and THCA (Figure 6D, P < 0.05), and its high expression is a risk factor in these tumor types (HR>1). KM-OS analysis showed that among the patients of ACC, BLCA, GBM, HNSC, KIRC, LGG, MESO, PAAD, STAD and thyroid carcinoma (THCA), those who expressed higher levels of *THBS1-IT1* had shorter survival time; in addiction, it also demonstrated a negative correlation was observed between *THBS1-IT1* expression level and outcome in ACC, BLCA, GBM, HNSC, KIRC, LGG and PAAD (Supplementary Figure 7 log-rank test P < 0.05). Cox-DSS data revealed that low *THBS1-IT1* expression had relative better prognosis in patients of ACC, BLCA, GBM, HNSC, KIRC, LGG, ovarian serous cystadenocarcinoma (OV), PAAD and STAD (Supplementary Figure 8, HR > 1, P < 0.05). KM-DSS displayed a correlation between *THBS1-IT1* and poor prognosis in ACC, BLCA, GBM, HNSC, KIRC LGG and PAAD patients (Supplementary Figure 9, log-rank test P<0.05). Both Cox and KM survival analysis showed that high expression of *THBS1-IT1* in LUAD, LUSC and sarcoma (SARC) was associated with shorter DFI (Supplementary Figures 10, 11). Forest plots showed upper expression of *THBS1-IT1* was associated with poor PFI in BLCA, GBM, HNSC, KIRC, LGG, LUAD, LUSC, PAAD, SARC and uveal melanoma (UVM) (Supplementary Figure 12). The same result was also found in GBM, LGG, HNSC, KIRC, LUSC, PAAD, LUAD and UVM patients by KM analysis (Supplementary Figure 13). Taken together, these survival analyses lead to a consistent conclusion that high expression of *THBS1-IT1* in tumors is related with poor outcome.

### Correlation of *THBS1-IT1* expression with clinical phenotypes in pan-cancer

As *THBS1-IT1* is an aging-related lncRNA, we further investigated the association between age and *THBS1-IT1* expression levels in pan-cancer. We found that elder patients (≥ 65 years) of BLCA, CESC, LAML, SARC, and THYM expressed higher *THBS1-IT1* than patients of < 65 years; on the contrary, elder UCEC patients expressed lower levels (Figure 6E). The relevance of tumor stage and *THBS1-IT1* expression was explored and we found that *THBS1-IT1* was significantly correlated with tumor stage in 13 types of cancer such as BLCA, KIRC and THCA (Figure 6F).

### Relationship between *THBS1-IT1* expression and the tumor microenvironment and tumor immune cell infiltration

Tumor immune microenvironment (TME) and tumor immune cell infiltration (TICI) are closely related to clinical outcome, they played as critical roles in tumor occurrence, tumor progression and cancer therapeutic response [34–36]. To further test the prognostic potential of *THBS1-IT1* in pan-cancer, the ESTIMATE algorithm [31] was used to calculate the stromal and immune cell scores in 33 types of cancer. It showed that *THBS1-IT1* was positively correlated with immune scores (r > 0.5, P < 0.001) in esophageal carcinoma (ESCA), KICH and pheochromocytoma and paraganglioma (PCPG) (Supplementary Figure 14). *THBS1-IT1* also exhibited strong connection with stromal scores in BLCA, KICH, ESCA, LUSC, READ, PAAD, MESO, OV, PCPG and STAD by pan-cancer analysis (r > 0.5, P < 0.001) (Supplementary Figure 15). In addition, *THBS1-IT1* is related with the infiltration of 6 immune cell types including B cells, Macrophages, CD4+ T cells, CD8+ T cells, Neutrophils and Dendritic cells in ESCA, liver hepatocellular carcinoma (LIHC) and LUSC (r > 0.3, P < 0.001) (Supplementary Figure 16). In the majority of the 33 tumor types, degree of macrophage infiltration was correlated with *THBS1-IT1* (r > 0.4, P < 0.001).

### Correlations of *THBS1-IT1* expression levels with tumor mutation burden and tumor microsatellite instability

Increasing evidence has implicated that tumor mutation burden (TMB) and tumor microsatellite instability (MSI) are potential markers associated with the efficacy of immunotherapy and/or chemotherapy and thus involving with tumor survival [37, 38]. To investigate the correlations between *THBS1-IT1* expression levels and TMB/MSI, Spearman’s rank correlation coefficient analysis was applied. Our results demonstrated that *THBS1-IT1* is related with TMB in 7 types of tumors including BLCA, BRCA, GBM, LIHC, LUSC, STAD and THYM (Supplementary Figure 17, P<0.05). In ESCA, HNSC, PRAD, STAD and THCA, *THBS1-IT1* was related to MSI (Supplementary Figure 18, P<0.05).

## DISCUSSION

Over the past decades, great efforts have been made to propel a better understanding of the driving forces that lead to aging and cancer [39]. To discipline the vital and complex links between aging, cellular senescence and cancer, the prognostic power of 8 candidate aging related lncRNAs (CarLncs) in KIRC were evaluated. Among these CarLncs, *THBS1-IT1*, *LEF1-AS1-201* and *WDR11-DT-201* showed significant variations between KIRC and tumor-adjacent tissues in TCGA data while *THBS1-AS1* and *DCHS1-AS1* displayed non-significant changes. *DCHS1-AS1* is sequence conserved and transcribed from the antisense of dachsous cadherin-related (*DCHS1*) [8]. Although the function of these dysregulated lncRNAs in KIRC remains to be explored, our results summarized a broad overview of their expression patterns in KIRC, which provided new insights into aging, cancer and lncRNA studies. Comparing with previous model that utilized m6A-Related lncRNA as signatures [14], we estimated the predictive power of 8 aging related lncRNAs in KIRC prognosis via a series of analyses, which enhanced the understanding of the associations between aging and cancers.

*THBS1-IT1* and *THBS1-AS1* are two lncRNAs that separately transcribed from the intronic and antisense regions of protein coding gene Thrombospondin 1 (*THBS1)*. THBS1 is a matricellular protein that has been shown to accelerate the production and modulation of relative oxidative stress (ROS) in vasoreactivity and in the peripheral circulation, making it an aging marker that contributes to the aging progress [40]. In addition, increasing studies suggest that *THBS1* is a prognostic biomarker of cancers and/or plays complex roles in various cancer types, such as glioblastoma [41, 42], papillary thyroid cancer [43], gastric cancer [44, 45], esophageal squamous cell carcinoma [46], and acute myeloid leukemia [47]. It is well documented that many lncRNAs can *in cis* control the expression of their nearby genes [48]*.* Previous study found that *THBS1-IT1* and *THBS1-AS1* are anti-aging lncRNAs and their expression was strongly correlated with the nearest gene *THBS1*, negatively or positively, suggesting they may be involved in the regulation of *THBS1* expression [8]. The faceted functions of *THBS1* in both aging and cancers as well as the regulation between *THBS1-IT1* and ER-stress/p53 signaling, we hypothesized *THBS1-IT1* may act as prognostic biomarkers in not only KIRC but also in a pan-cancer manner. We surprisingly found that *THBS1-IT1* was dysregulated in 12 tumors and its high expression is associated with poor prognosis in multiple tumors, which suggests *THBS1-IT1* may act as a predominant factor involved in both aging and cancer regulatory empires although the deeper mechanisms remain to be explored.

## CONCLUSIONS

In this study, we seek for providing new connections between aging and cancers from the perspective of lncRNAs. An integrated regulatory network of CarLncs, miRNAs and mRNAs was constructed. Five aging-related signature genes within the network were identified by analyses such as SVM and RF, and they were utilized for developing the aging related model of KIRC. The robustness and efficiency of our model was evaluated by Nomogram, which suggested that LncAging_scores is a powerful predictive model for KIRC prognosis. Our next-step pan-cancer analysis found that one of the signature genes, *THBS1-IT1*, is a potential prognostic biomarker for multiple cancers. A series of survival analysis consistently refers to a strong correlation between its high expression and poor outcome.

## Supplementary Material

Supplementary Figures

Supplementary Table 1

Supplementary Table 2

Supplementary Table 3

Supplementary Table 4
