# Supplementary material for: Prognostic model of kidney renal clear cell carcinoma using aging-related long noncoding RNA signatures identifies THBS1-IT1 as a potential prognostic biomarker for multiple cancers
Source: Aging (Albany NY). 2023 Sep 13;15(17):8630–63. doi: 10.18632/aging.204949 (PMC10522375; doi:10.18632/aging.204949)
Supplement: Supplementary Figures [file aging-15-204949-s001.pdf]

SUPPLEMENTARY FIGURES

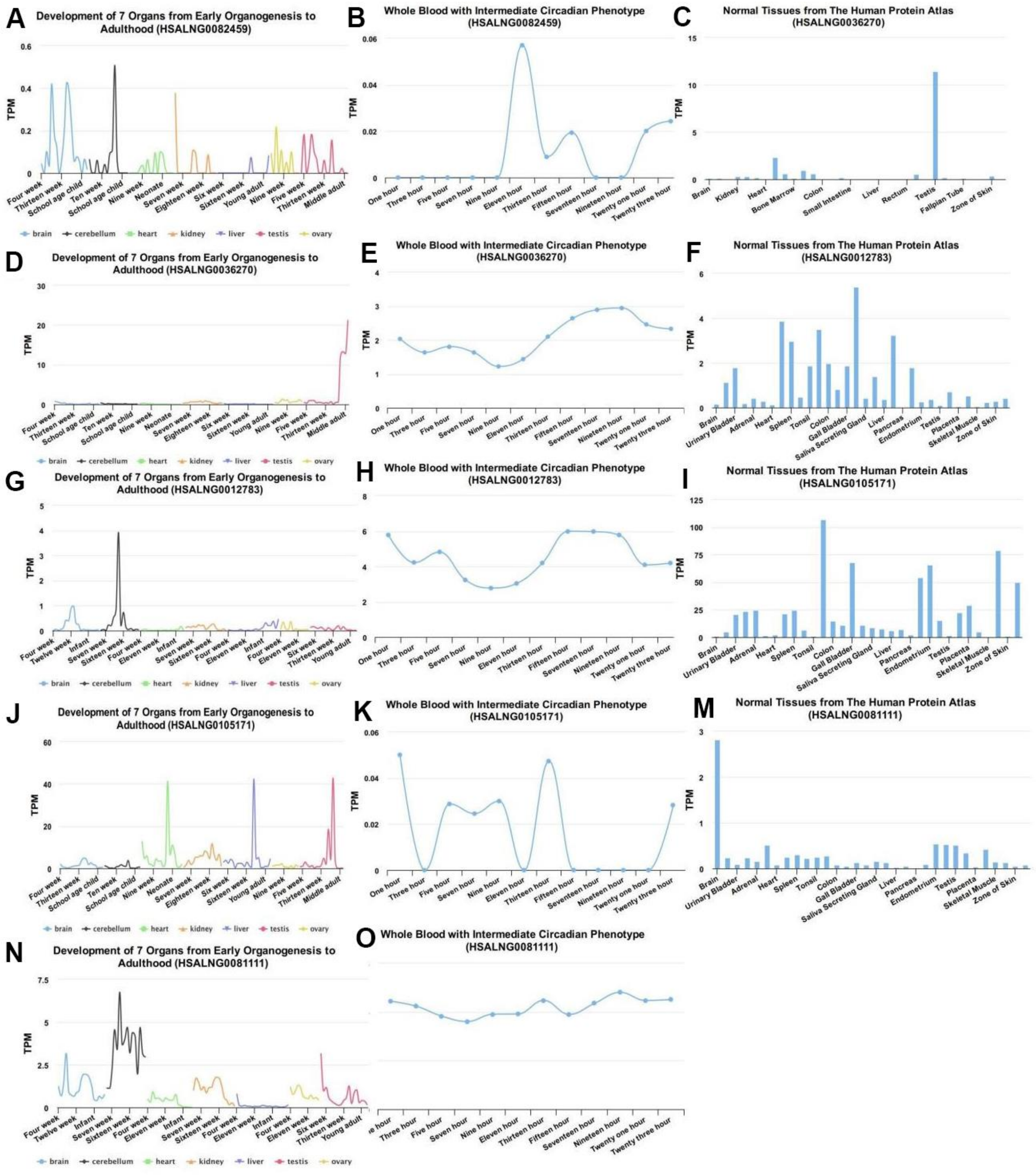

Supplementary Figure 1. Dynamic expression patterns of five Car-Lncs include DCHS1-AS1 (**A**, **B**), LEF1-AS1-201 (**C**–**E**), LINC01871 (**F**–**H**), THBS1-IT1 (**I**–**K**) and WDR11-DT-201 (**M**–**O**).

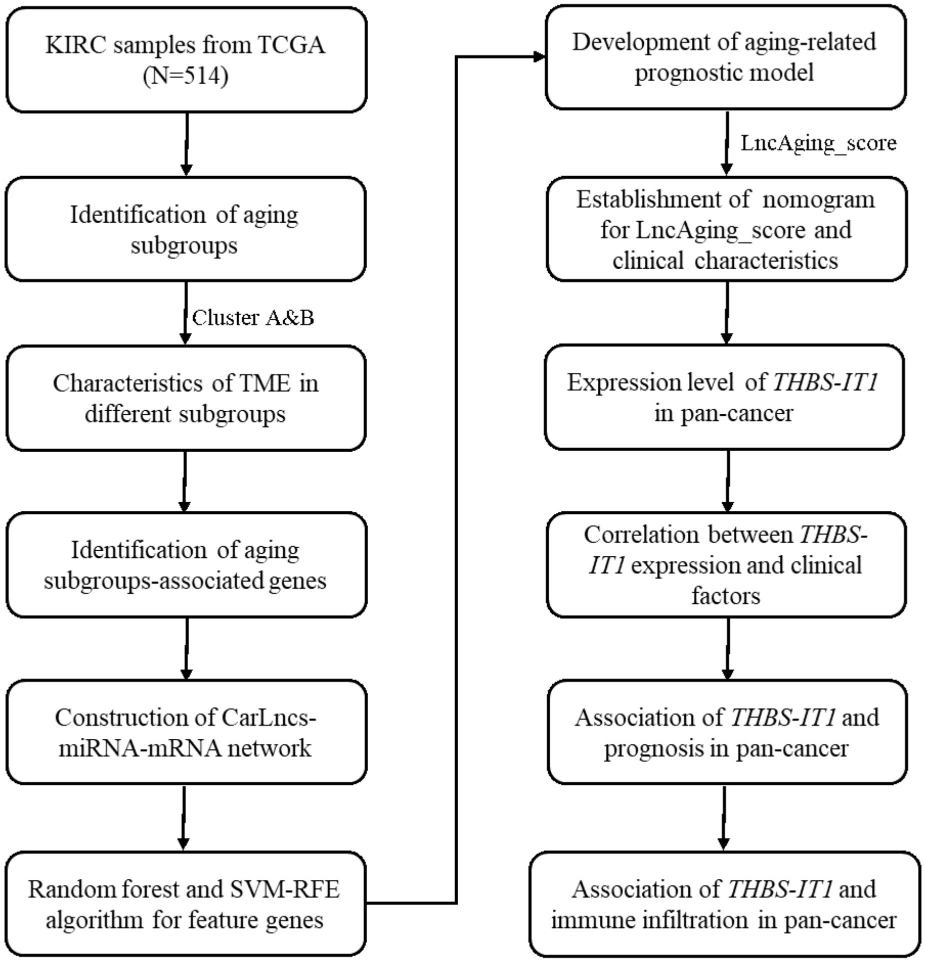

Supplementary Figure 2. The entire analytical process of the study.

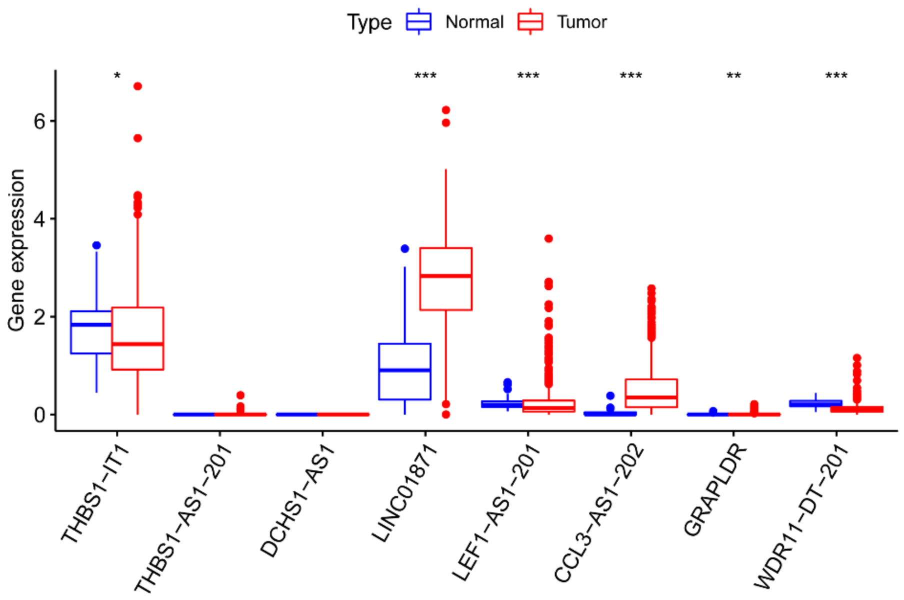

Supplementary Figure 3. Comparison of expression levels of 8 CarLncs between tumor and adjacent tissues of KIRC patients.

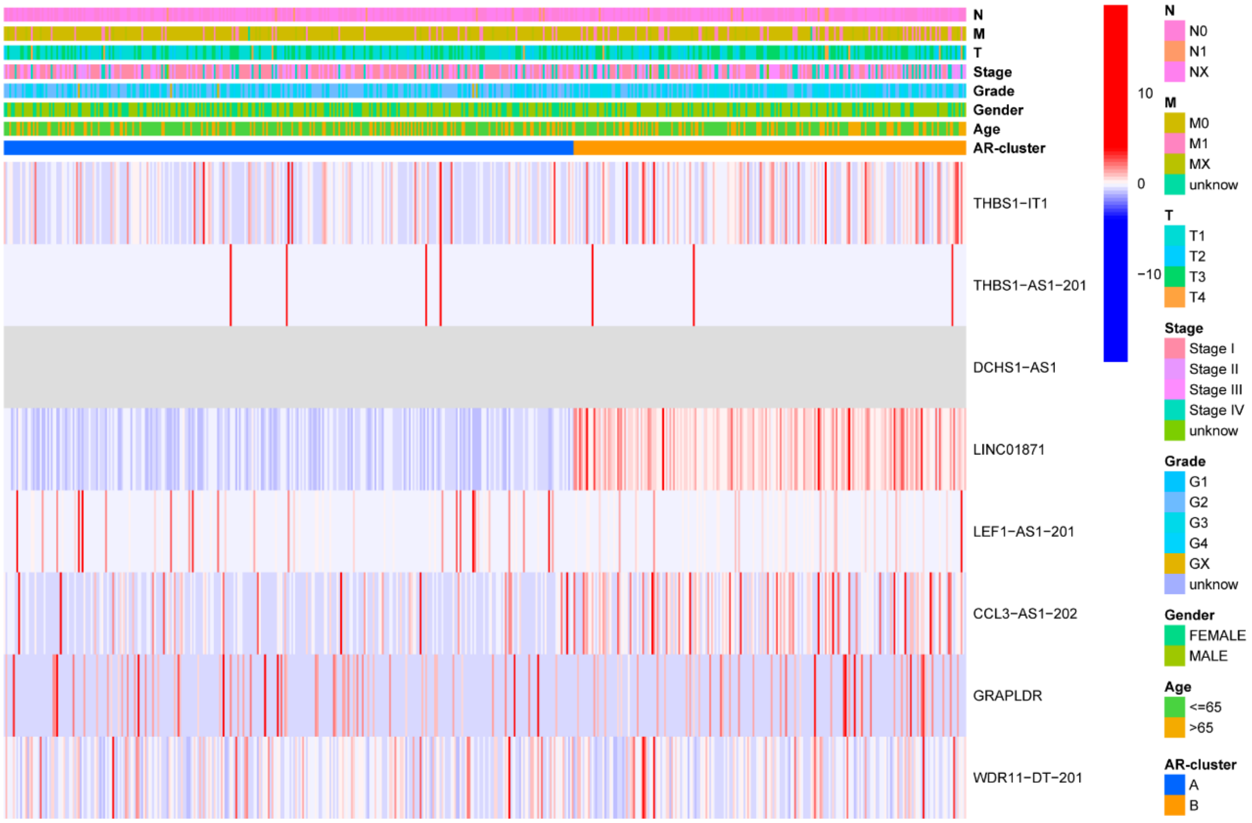

Supplementary Figure 4. Comparisons of the expression level of 8 CarLncs and clinicopathological variables.

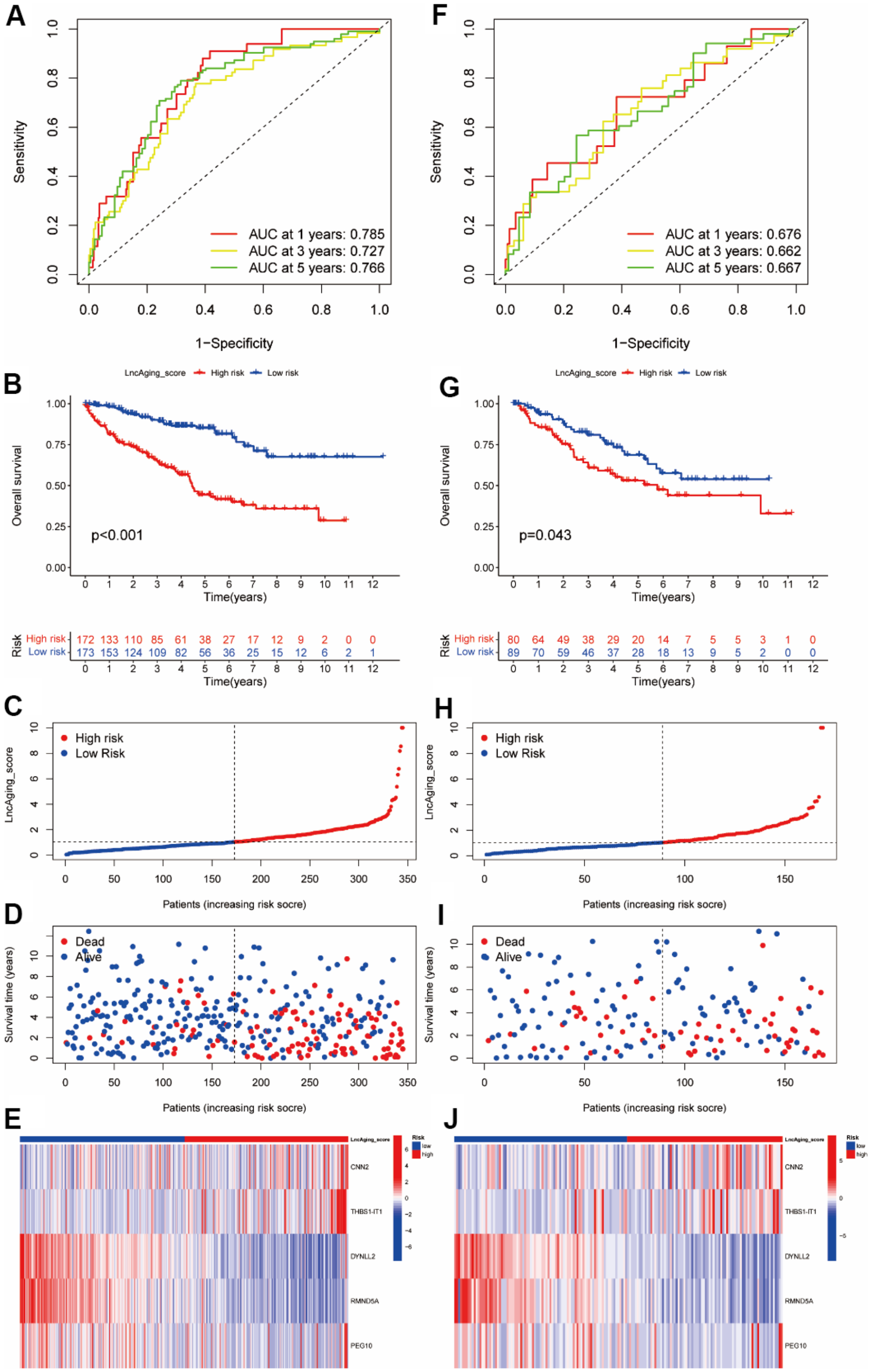

Supplementary Figure 5. Validation of LncAging_score in training (**A**–**E**) and validation (**F**–**J**) cohort. (**A**, **F**) ROC curves to predict the sensitivity and specificity of 1-, 3-, and 5-year survival according to the LncAging_score. (**B**, **G**) KM analysis of the OS between the two groups. (**C**, **D**, **H**, **I**) The ranked dot plot indicates the LncAging_score distribution and scatter plot presenting the patients' survival status. (**E**, **J**) Expression patterns of 5 selected prognostic genes in high- and low-risk groups.

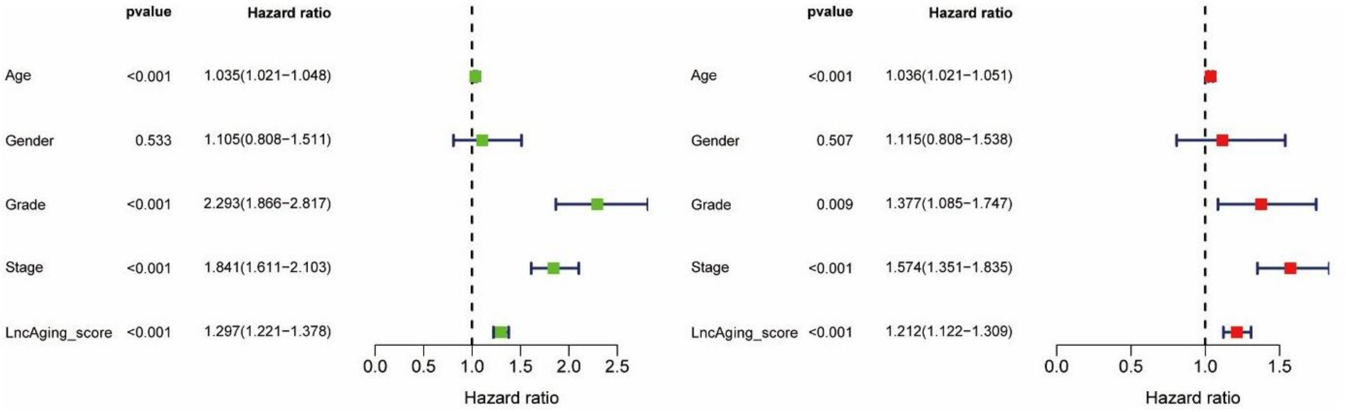

Supplementary Figure 6. The independent prognosis analysis of LncAging_score and clinicopathological variables in KIRC. Univariate (left panel) and multivariate (right panel) analyses showed the prognostic value of the LncAging_score in the entire cohort.

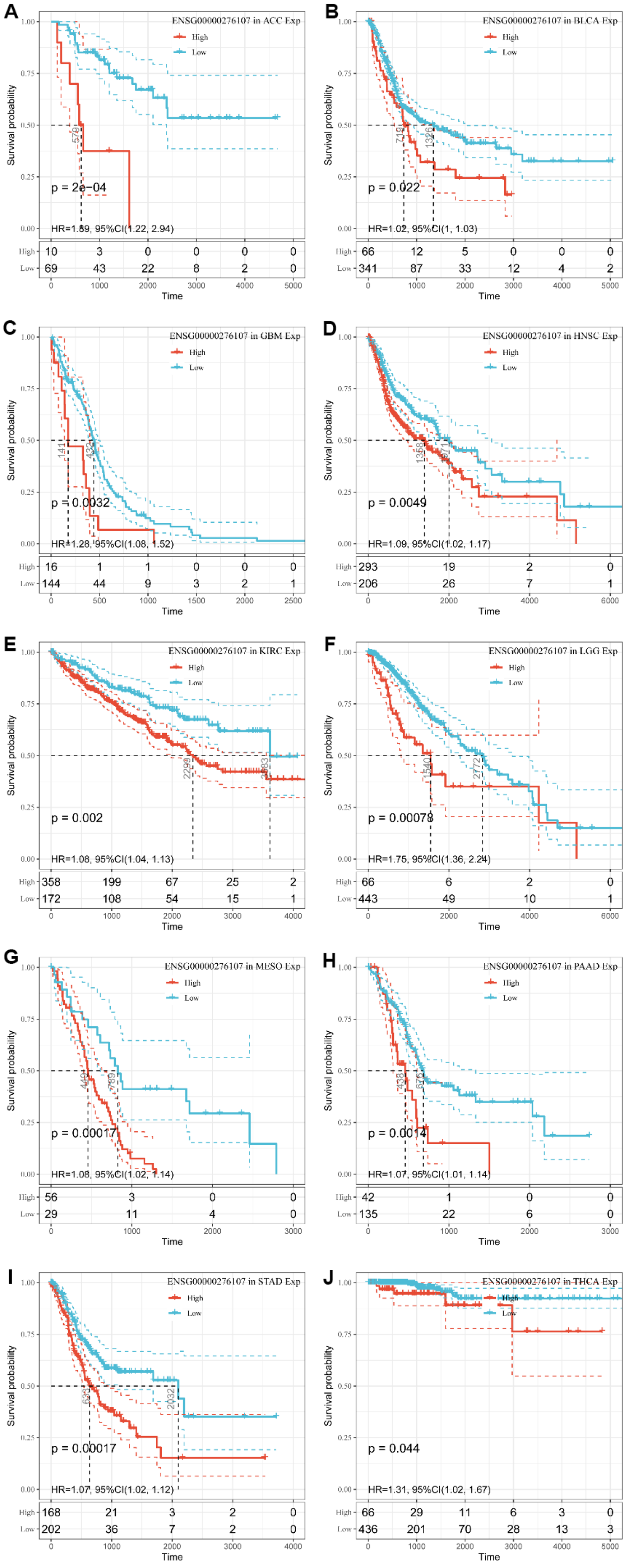

Supplementary Figure 7. Kaplan-Meier analysis of the association between *THBS1-IT1* (ensemble ID: ENSG00000276107) expression and OS. (**A**) ACC; (**B**) BLCA; (**C**) GBM; (**D**) HNSC; (**E**) KIRC; (**F**) LGG; (**G**) MESO; (**H**) PAAD; (**I**) STAD; (**J**) THCA.

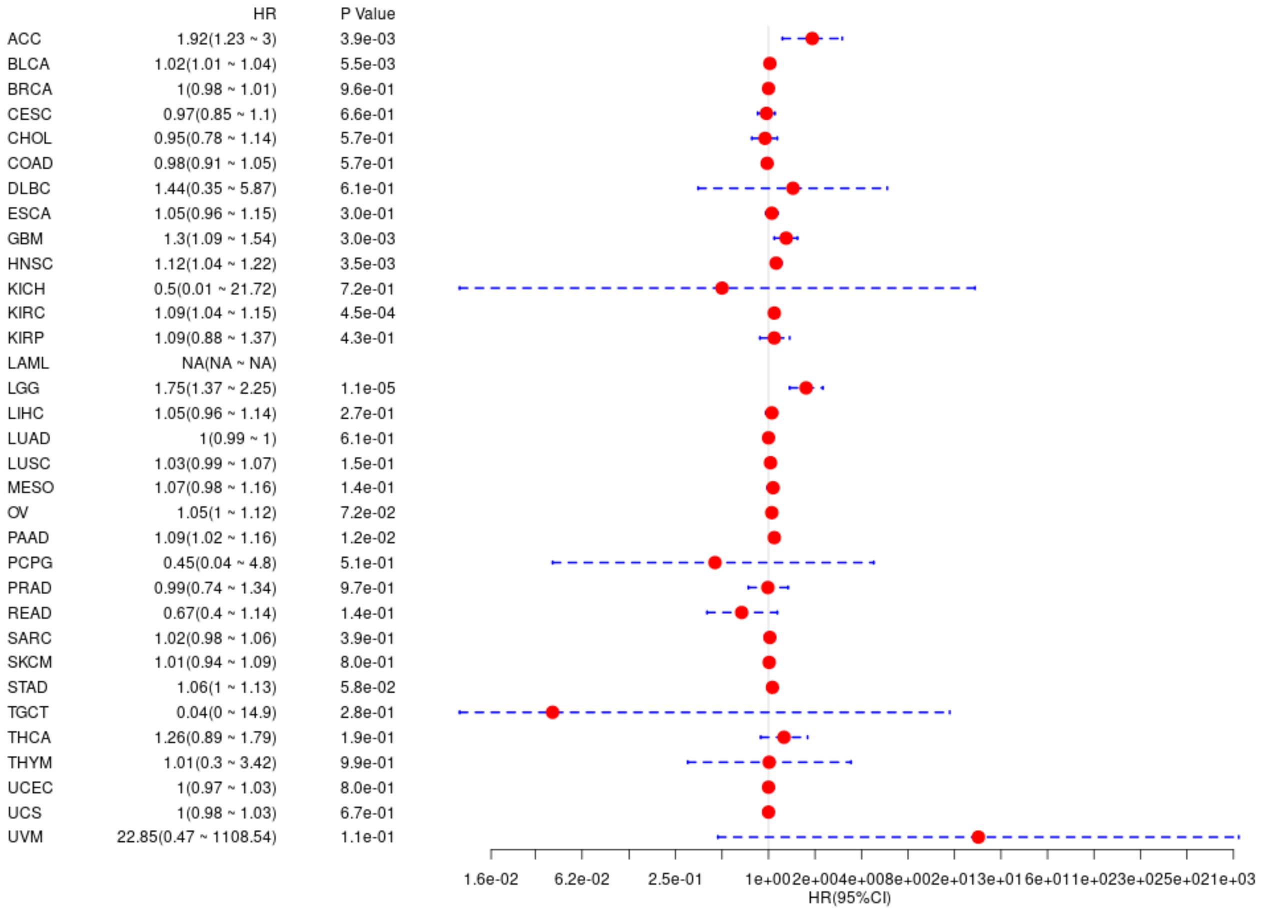

Supplementary Figure 8. Forest plot of association of *THBS1-IT1* with disease-specific survival (DSS) for 33 types of tumors.

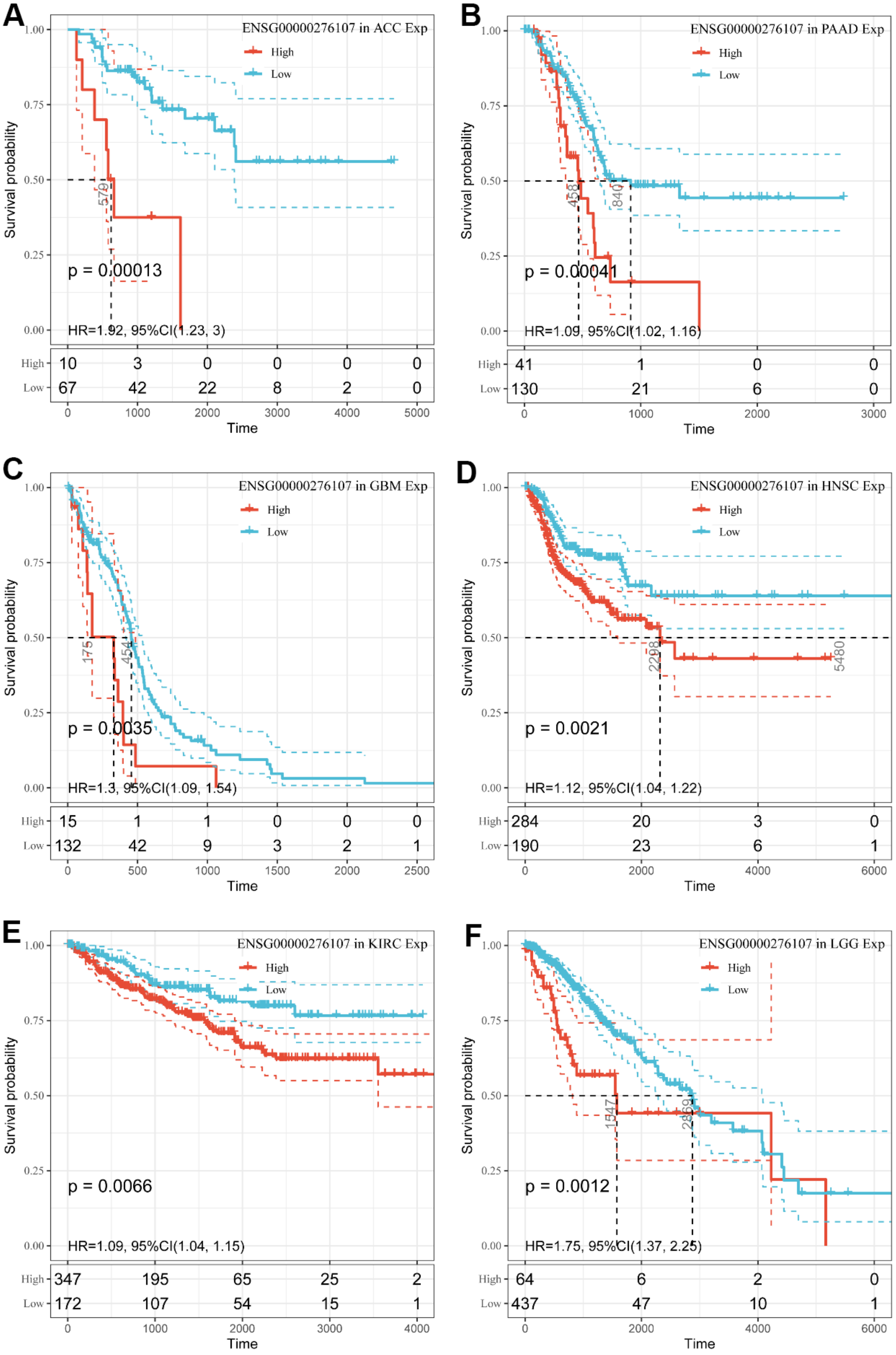

Supplementary Figure 9. Kaplan-Meier analysis of the association between *THBS1-IT1* (ensemble ID: ENSG00000276107) expression and DSS. (**A**) ACC; (**B**) PAAD; (**C**) GBM; (**D**) HNSC; (**E**) KIRC; (**F**) LGG.

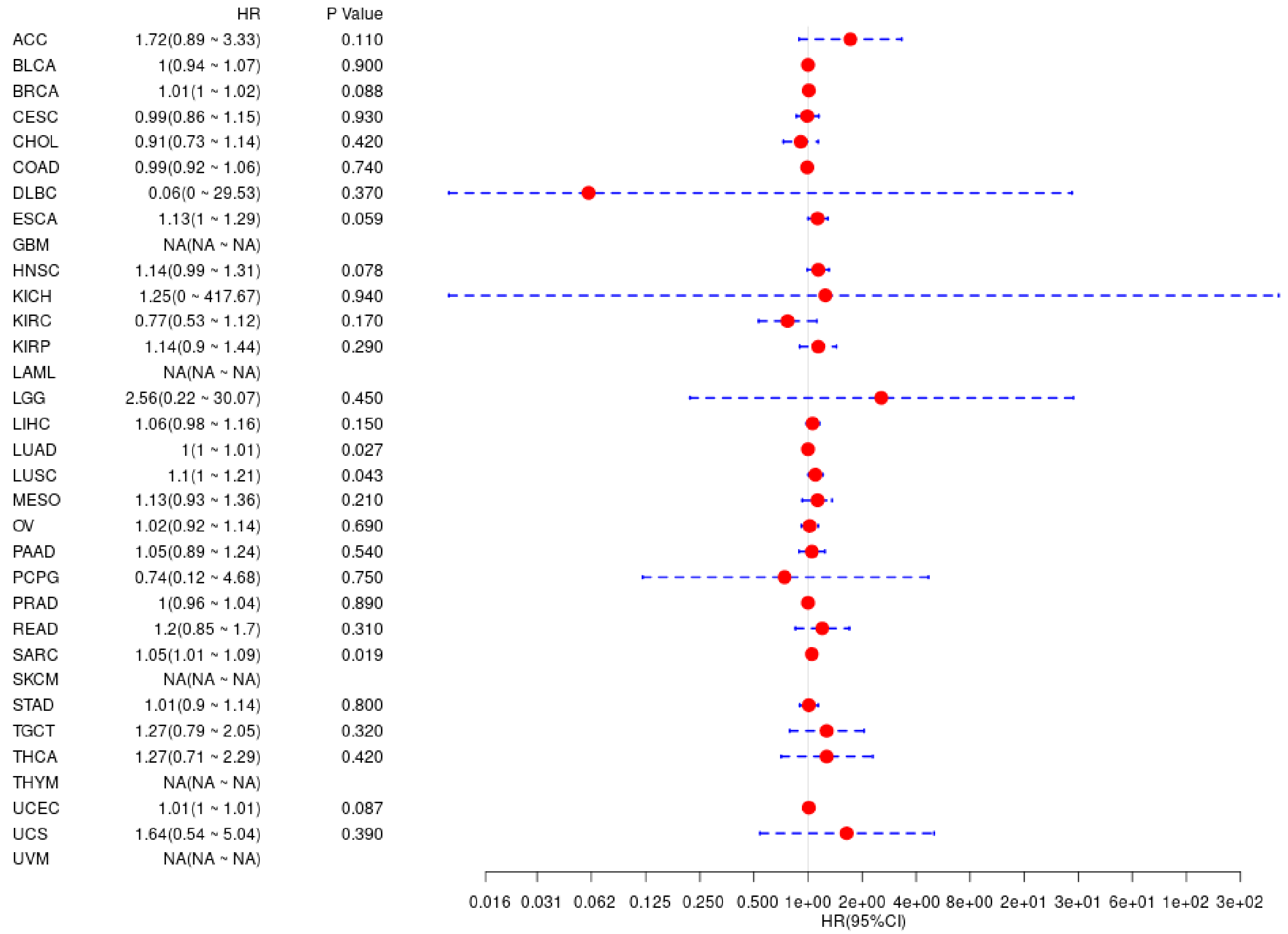

Supplementary Figure 10. Forest plot of association of *THBS1-IT1* with disease-free interval (DFI) for 33 types of tumors.

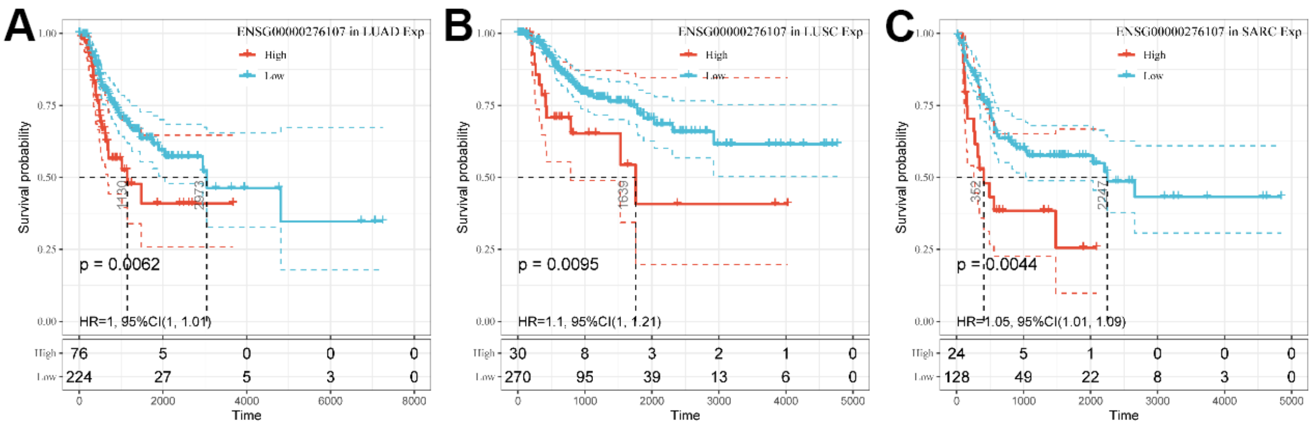

Supplementary Figure 11. Kaplan-Meier analysis of the association between *THBS1-IT1* (ensemble ID: ENSG00000276107) expression and DFI in LUAD (**A**), LUSC (**B**) and SARC (**C**).

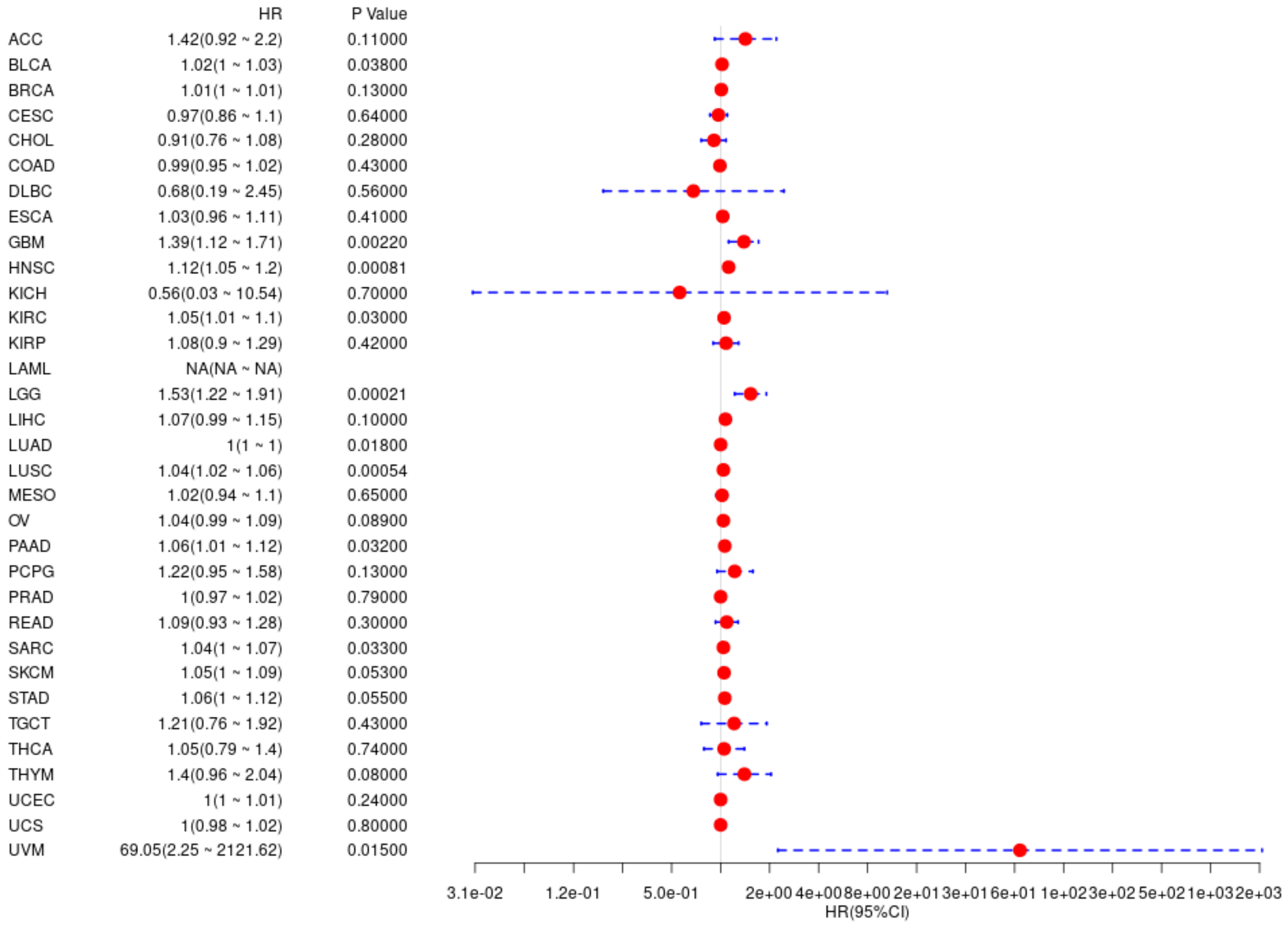

Supplementary Figure 12. Forest plot of association of *THBS1-IT1* with progression-free interval (PFI) for 33 types of tumors.

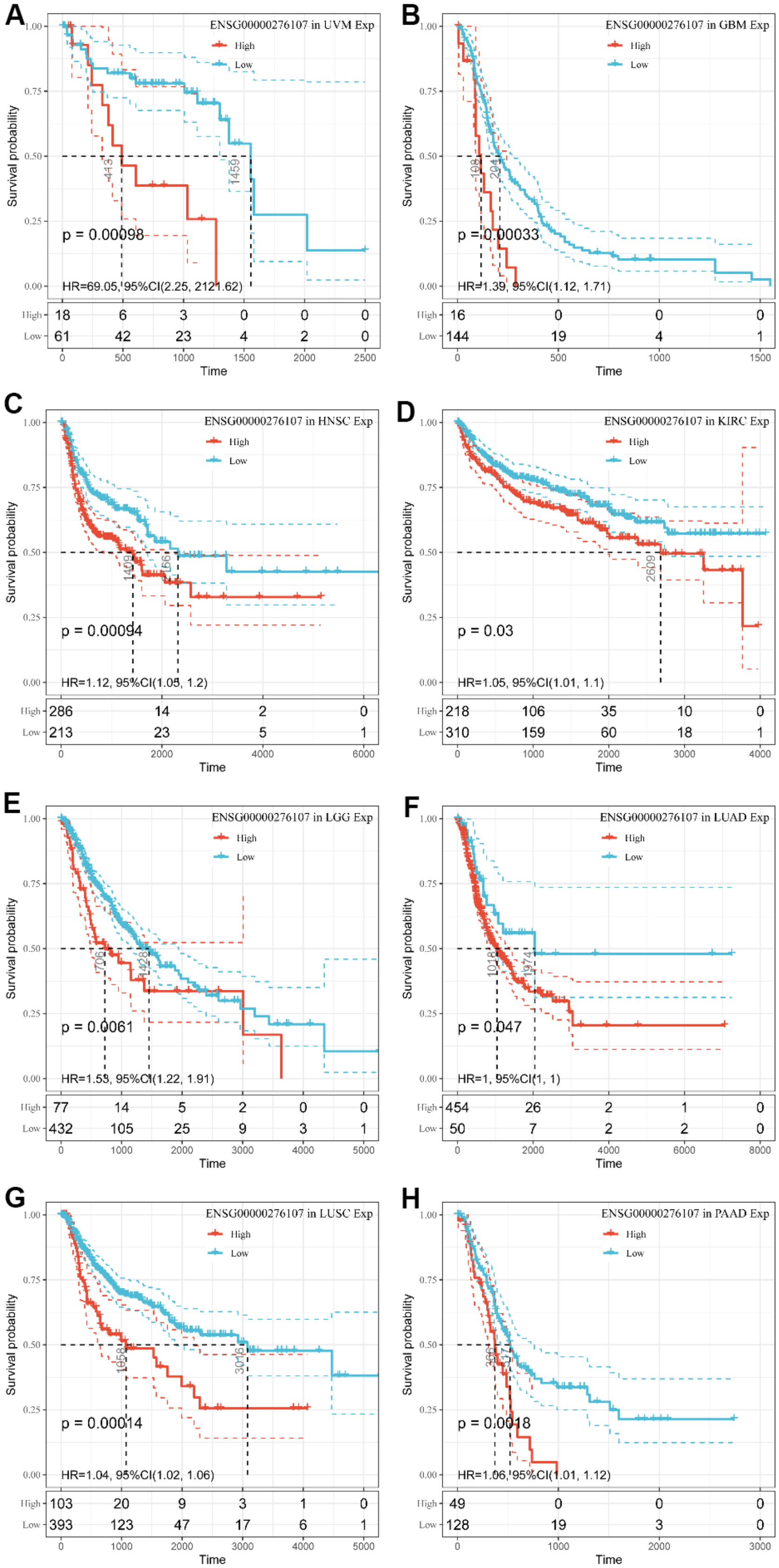

Supplementary Figure 13. Kaplan-Meier analysis of the association between *THBS1-IT1* (ensemble ID: ENSG00000276107) expression and PFI. (**A**) UVM; (**B**) GBM; (**C**) HNSC; (**D**) KIRC; (**E**) LGG; (**F**) LUAD; (**G**) LUSC; (**H**) PAAD.

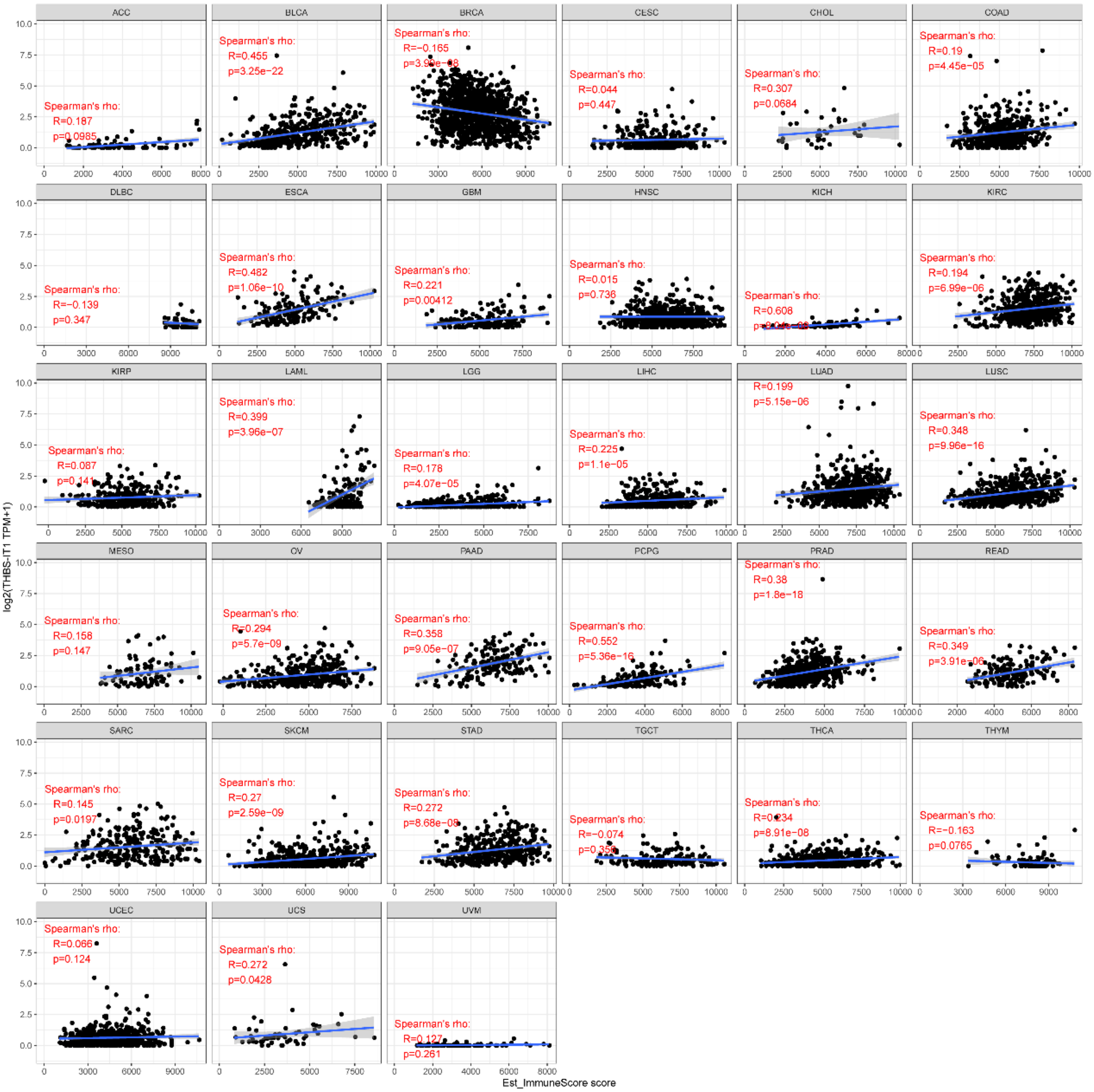

Supplementary Figure 14. Correlation between *THBS1-IT1* expression and immune scores in 33 types of tumors.

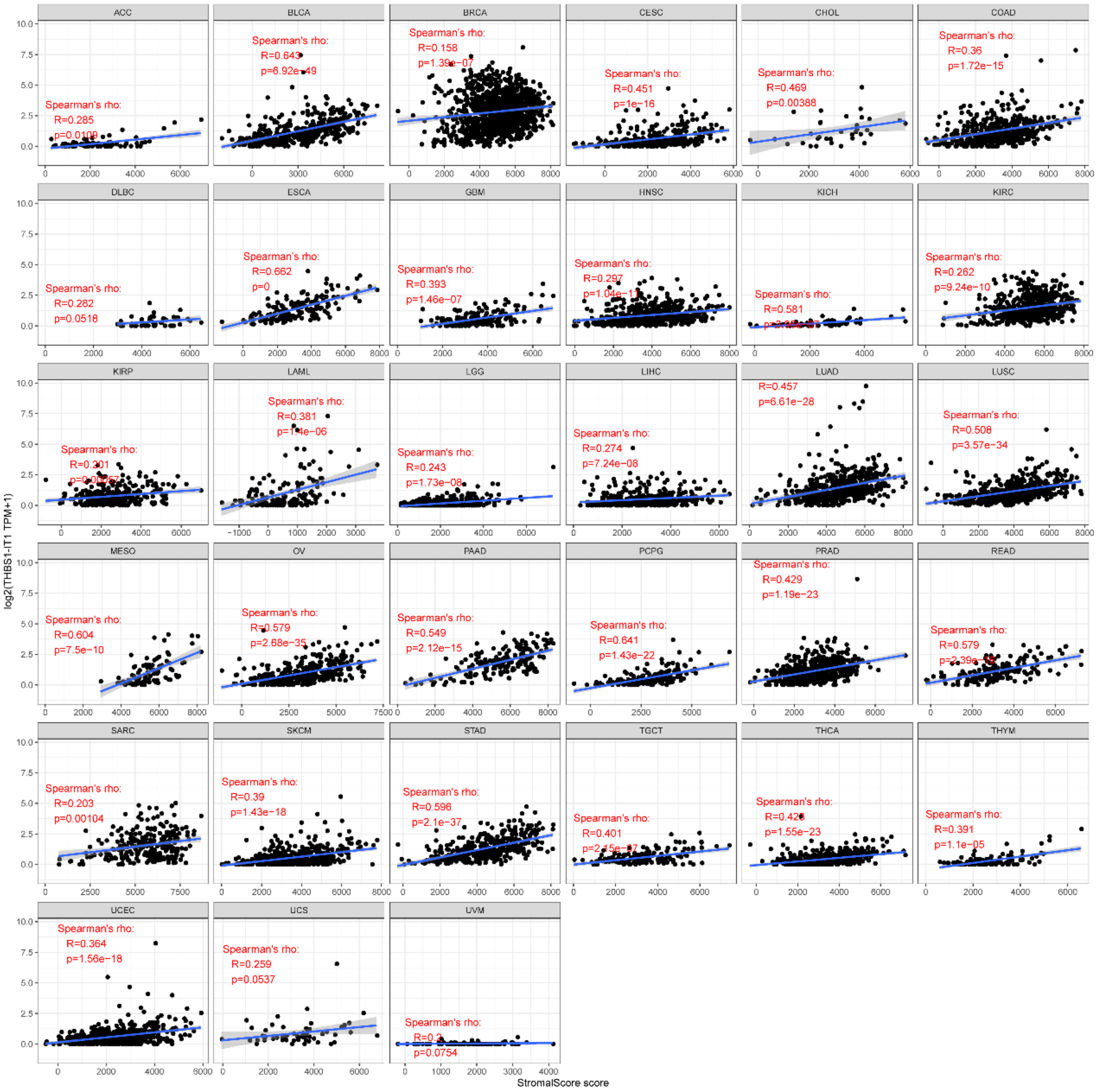

Supplementary Figure 15. Correlation between *THBS1-IT1* expression and stromal scores in 33 types of tumors.

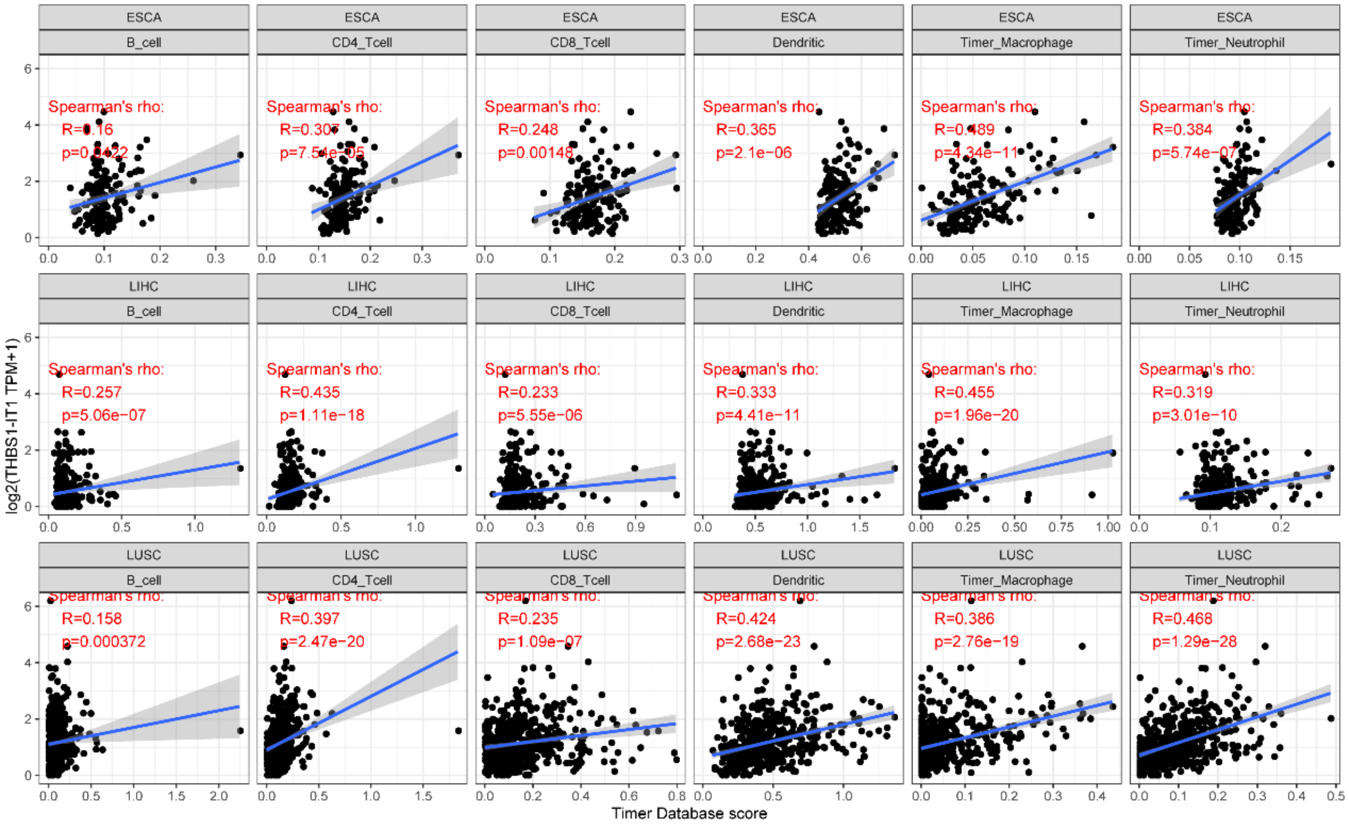

Supplementary Figure 16. Correlation between TREM2 expression and immune cell infiltration in ESCA, LIHC and LUSC.

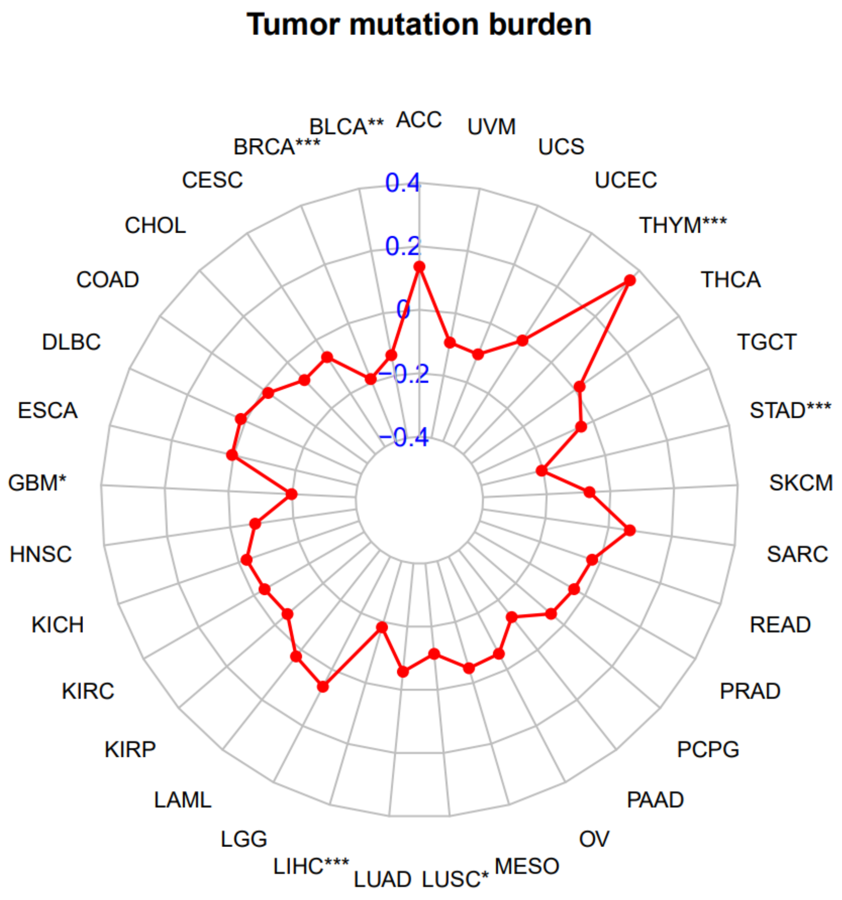

Supplementary Figure 17. Associations between *THBS1-IT1* expression and tumor mutational burden (TMB). The correlation coefficient *p < 0.05, **p < 0.01, and ***p < 0.001.

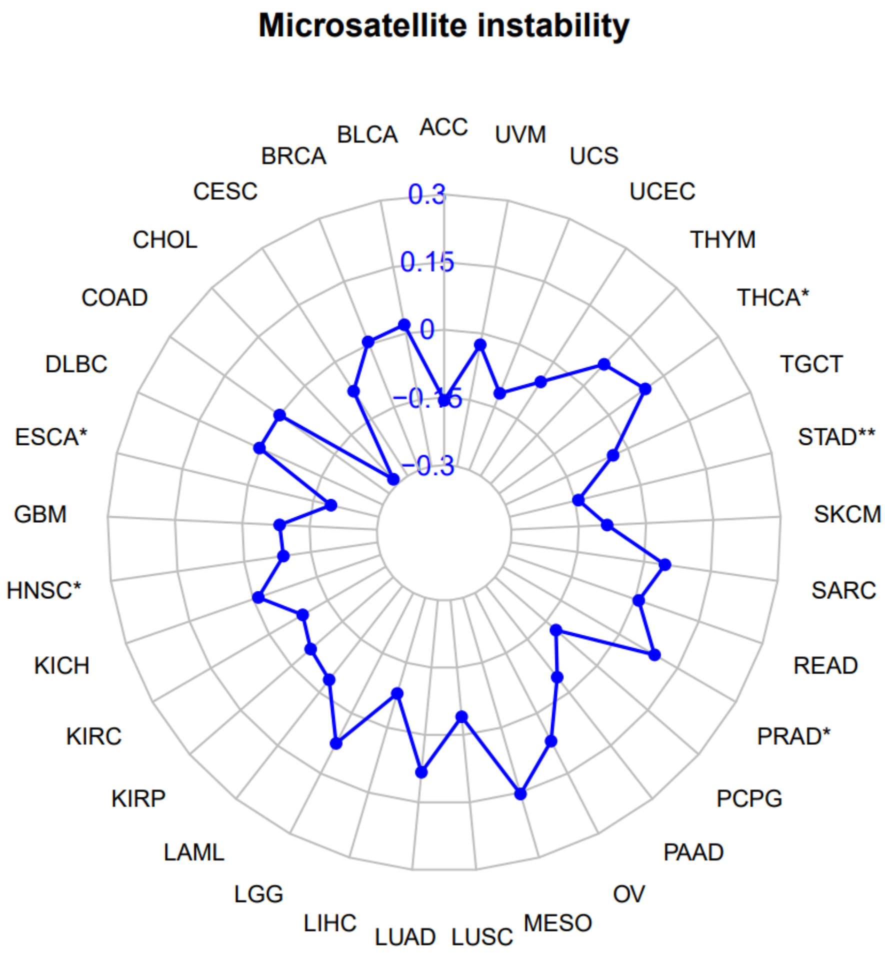

Supplementary Figure 18. Associations between *THBS1-IT1* expression and microsatellite instability (MSI). The correlation coefficient *p < 0.05, **p < 0.01, and ***p < 0.001.
