# Supplementary material for: Prognostic model of kidney renal clear cell carcinoma using aging-related long noncoding RNA signatures identifies THBS1-IT1 as a potential prognostic biomarker for multiple cancers
Source: Aging (Albany NY). 2023 Sep 13;15(17):8630–63. doi: 10.18632/aging.204949 (PMC10522375; doi:10.18632/aging.204949)
Supplement: Supplementary Table 1 [file aging-15-204949-s002.pdf]

SUPPLEMENTARY TABLE

Supplementary Table 1. Survival/clinicopathological information of 514 KIRC patients.

Characteristics	Frequency (N=514)	Percentage (%)
**Age**		
<65	325	63.2
≥65	189	36.8
**Gender**		
FEMALE	180	35.0
MALE	334	65.0
**Grade**		
G1	13	2.5
G2	217	42.2
G3	201	39.1
G4	75	14.6
GX	5	1.0
unknown	3	0.6
**Pathologic stage**		
Stage I	252	49.0
Stage II	55	10.7
Stage III	122	23.7
Stage IV	82	16.0
unknown	3	0.6
**Survival status**		
Alive	343	66.7
Dead	171	33.3
**T stage**		
T1	258	50.2
T2	67	13.0
T3	178	34.6
T4	11	2.1
**M stage**		
M0	404	78.6
M1	78	15.2
MX	30	5.8
unknown	2	0.4
**N stage**		
N0	228	44.4
N1	16	3.1
Nx	270	52.5
