# Supplementary material for: Prognostic model of kidney renal clear cell carcinoma using aging-related long noncoding RNA signatures identifies THBS1-IT1 as a potential prognostic biomarker for multiple cancers
Source: Aging (Albany NY). 2023 Sep 13;15(17):8630–63. doi: 10.18632/aging.204949 (PMC10522375; doi:10.18632/aging.204949)
Supplement: Supplementary Table 4 [file aging-15-204949-s005.pdf]

Supplementary Table 4. Comparison of clinical variables between the training set and validation set.

Covariates	Entire set	Training set	Validation set	P-value
**Age**				**0.7477**
<=65	341(66.34%)	231(66.96%)	110(65.09%)	
>65	173(33.66%)	114(33.04%)	59(34.91%)	
**Gender**				**0.7954**
FEMALE	180(35.02%)	119(34.49%)	61(36.09%)	
MALE	334(64.98%)	226(65.51%)	108(63.91%)	
**Grade**				**0.9163**
G1	13(2.53%)	8(2.32%)	5(2.96%)	
G2	217(42.22%)	148(42.9%)	69(40.83%)	
G3	201(39.11%)	132(38.26%)	69(40.83%)	
G4	75(14.59%)	51(14.78%)	24(14.2%)	
unknow	8(1.56%)	6(1.74%)	2(1.18%)	
**Stage**				**0.3022**
Stage I	252(49.03%)	174(50.43%)	78(46.15%)	
Stage II	55(10.7%)	38(11.01%)	17(10.06%)	
Stage III	122(23.74%)	73(21.16%)	49(28.99%)	
Stage IV	82(15.95%)	57(16.52%)	25(14.79%)	
unknow	3(0.58%)	3(0.87%)	0(0%)	
